# Mechanistic Insights and Catalytic Efficiency of a 2,2′‐Bipyridine–Coordinated Peroxidovanadium Complex as a Haloperoxidase Biomimetic

**DOI:** 10.1002/cplu.202500444

**Published:** 2025-09-01

**Authors:** Lucas G. Fachini, Enzo V. S. Elisandro, Gabriel B. Baptistella, Rúbia C. R. Bottini, Matteo Briganti, Giovana G. Nunes, Eduardo L. de Sá

**Affiliations:** ^1^ Departamento de Química Universidade Federal do Paraná, Centro Politécnico Jardim das Américas 81530–900 Curitiba PR Brazil; ^2^ Departamento de Química e Biologia Universidade Tecnológica Federal do Paraná Rua Deputado Heitor de Alencar Furtado, Ecoville 81280–340 Curitiba PR Brazil; ^3^ Dipartimento di Chimica Ugo Schiff and INSTM RU Università degli Studi di Firenze Via della Lastruccia 3–13 50019 Sesto Fiorentino–FI Italy

**Keywords:** ab initio calculations, halogenation mechanism, haloperoxidase, organic substrates, peroxidovanadium complex

## Abstract

The peroxidovanadium(V) complex [V^V^O(O_2_)(HO_2_)(bpy)]·3H_2_O·0.5bpy (**1**), where bpy = 2,2′‐bypiridine, featuring *η* ^2^‐coordinated peroxide and hydroperoxide ligands, is reported as an efficient functional model of vanadium haloperoxidases (VHPOs). Structural and spectroscopic analyses indicate similarities between **1** and VHPO active sites, including peroxide ligand protonation. Mechanistic studies employing ab initio computational methods are based on the presence of [V^V^O(O_2_)(HO_2_)(bpy)] and its aqueous equilibrium species [V^V^O(O_2_)(HO_2_)(H_2_O)], in solutions of **1** (pH = 5.8). For each compound, two reaction pathways are explored for the oxidation of iodide and bromide: 1) direct HOX, where X = Br or I, formation through nucleophilic attack on the protonated *η* ^2^‐peroxide, affording Δ*G* ^‡ ^= 20.0–26.5 kcal mol^−1^ and 2) V–OX intermediate formation after the nucleophilic attack on the *η* ^2^‐peroxide resulting in Δ*G* ^‡ ^= 15.6–17.6 kcal mol^−1^. Catalyst regeneration via end‐on H_2_O_2_ coordination is exergonic (Δ*G *= −15.2 and −21.6 kcal mol^−1^), indicating sustainable turnover. Complex **1** catalyzes the oxidative bromination of phenol red with a rate constant of 990 ± 90 mol^−2 ^L^2 ^min^−1^ and achieves high‐yield halogenation of 8‐hydroxyquinoline (73 and 86% for 5,7‐dibromoquinolin‐8‐ol and 5,7‐diiodoquinolin‐8‐ol) in mild conditions (30 °C, pH 5.8). The results highlight **1** as an efficient catalyst, with potential applications in the pharmaceutical and agrochemical industries.

## Graphical abstract

A peroxidovanadium(V) complex mimics vanadium haloperoxidases for eco‐friendly halogenation of organic substrates. Density functional theory studies reveal two major reaction pathways for iodide/bromide oxidation. The catalyst offers high‐yield bromination/iodination of 8‐hydroxyquinoline (73/86%) under mild conditions (30 °C, pH 5.8), and it also oxidizes phenol red (990 ± 90 mol^−2^ L^2^ min^−1^) efficiently to bromophenol blue.

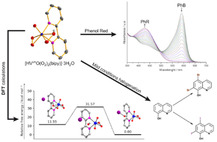

## 1 Introduction

Halogenation processes under mild conditions have attracted significant attention since they are a key strategy to enhance the biological activity and metabolic stability of organic molecules.^[^ ^1^ ^,^ ^2^ ^]^ In this context, organohalides are among the most important classes of compounds in organic chemistry, with applications in bioactive molecules, agrochemicals, and organic materials.^[^ ^1^ ^]^ A major drawback of organohalide production is the generation of environmentally harmful byproducts, such as Br_2_ and HBr.^[^ ^3^ ^,^ ^4^ ^]^ Therefore, the development of greener halogenation methods using reactants like hydrogen peroxide and molecular oxygen is highly desirable.^[^ ^3^ ^,^ ^5^ ^]^

Nature offers an efficient alternative through vanadium haloperoxidases (VHPOs), enzymes that catalyze halogenation in aqueous conditions using halides (X^−^) and hydrogen peroxide.^[^ ^6^ ^,^ ^7^ ^]^ These enzymes utilize a vanadate cofactor within a conserved active site to generate hypohalous acids.^[^ ^5^ ^,^ ^8^ ^,^ ^9^ ^]^ VHPOs function under mildly acidic conditions (pH from 4.5 to 6.0) and exhibit remarkable selectivity, making them attractive models for biomimetic catalysis.^[^ ^1^ ^,^ ^10^ ^]^ Despite their efficiency, VHPOs face limitations such as high production costs, a narrow substrate scope, and sensitivity to nonphysiological conditions.^[^ ^11^ ^]^ These challenges have driven interest in synthetic analogs that mimic their reactivity while offering enhanced stability and versatility.

Inspired by these enzymes, synthetic transition‐metal‐based functional models have been developed as potential catalysts for sustainable halogenation reactions.^[^ ^8^ ^,^ ^12^ ^]^ These biomimetic systems often employ vanadium, molybdenum, tungsten, or rhenium complexes, which form *η* ^2^‐peroxide intermediates, V^V^O(O_2_), analogous to those in the active sites of VHPOs.^[^ ^13^ ^,^ ^14^ ^]^ Over the years, several vanadium complexes have been explored for biomimetic halogenation, including oxidovanadium(V) species with multidentate ligands,^[^ ^14^ ^]^ mono‐ and binuclear oxidovanadium compounds,^[^ ^15^ ^]^ oxidovanadium(IV) complexes with porphyrin ligands,^[^ ^16^ ^]^ mixed‐valence binuclear oxidovanadium complexes,^[^ ^17^ ^]^ and peroxidovanadium complexes.^[^ ^18^ ^]^ This subject was recently reviewed and thoroughly discussed in the literature,^[^ ^1^ ^]^ however, a detailed understanding of their reactivity remains an open field. Advances in computational chemistry have provided deeper insights into the electronic structure of VHPO active sites and into the reaction pathways involved in the halogenation process, shedding light on key factors that govern their activity.^[^ ^15^ ^,^ ^19^ ^–^ ^21^ ^]^ Such knowledge is crucial for the rational design of catalysts with improved performance.

Herein, the peroxidovanadium complex [V^V^O(O_2_)(HO_2_)(bpy)]·3H_2_O·0.5bpy (bpy = 2,2’‐bipyridine) (**1**), first synthesized by Vuletić et al.^[^ ^22^ ^,^ ^23^ ^]^ [The cambridge crystallographic data centre deposition number: 1124110], was explored as a catalyst inspired by the active site of VHPOs. ^51^V NMR studies indicated the presence of two peroxidovanadium species in solution, [V^V^O(O_2_)(HO_2_)(bpy)] and [V^V^O(O_2_)(HO_2_)(H_2_O)], which could act as the catalytic species for bromination and iodination reactions of organic molecules. For experimental details, see the Supporting Information. Computational studies were conducted to propose possible reaction pathways, offering detailed mechanistic perspectives. Hence, two distinct reaction pathways, analogous to those observed in natural VHPOs,^[^ ^24^ ^,^ ^25^ ^]^ were evaluated using density functional theory (DFT) and the recently developed domain‐based local pair natural orbital (DLPNO)–CCSD(T) ab initio methods: Mechanism A, involving the direct formation of hypohalous acid (HOX), and Mechanism B, involving a vanadium‐bound hypohalide intermediate (V–OX).

Complex **1** was employed for the oxidative bromination of phenol red (PhR), a classical substrate for halogenation kinetics studies in homogeneous media. To further explore the catalytic potential of this peroxidovanadium complex, **1** was used for the halogenation of 8‐hydroxyquinoline, producing 5,7‐dibromoquinolin‐8‐ol^[^ ^26^ ^,^ ^27^ ^]^ and 5,7‐diiodoquinolin‐8‐ol,^[^ ^28^ ^,^ ^29^ ^]^ two commercial derivatives broadly used by the pharmaceutical industry. This study contributes to the rational design of sustainable catalysts, addressing the key steps involved in both enzymatic and synthetic oxidative halogenation of organic substrates promoted by peroxidovanadium complexes.

## 2 Results and Discussion

### 2.1 The Structure of 1 and VHPOs Correlations

**Figure 1:**
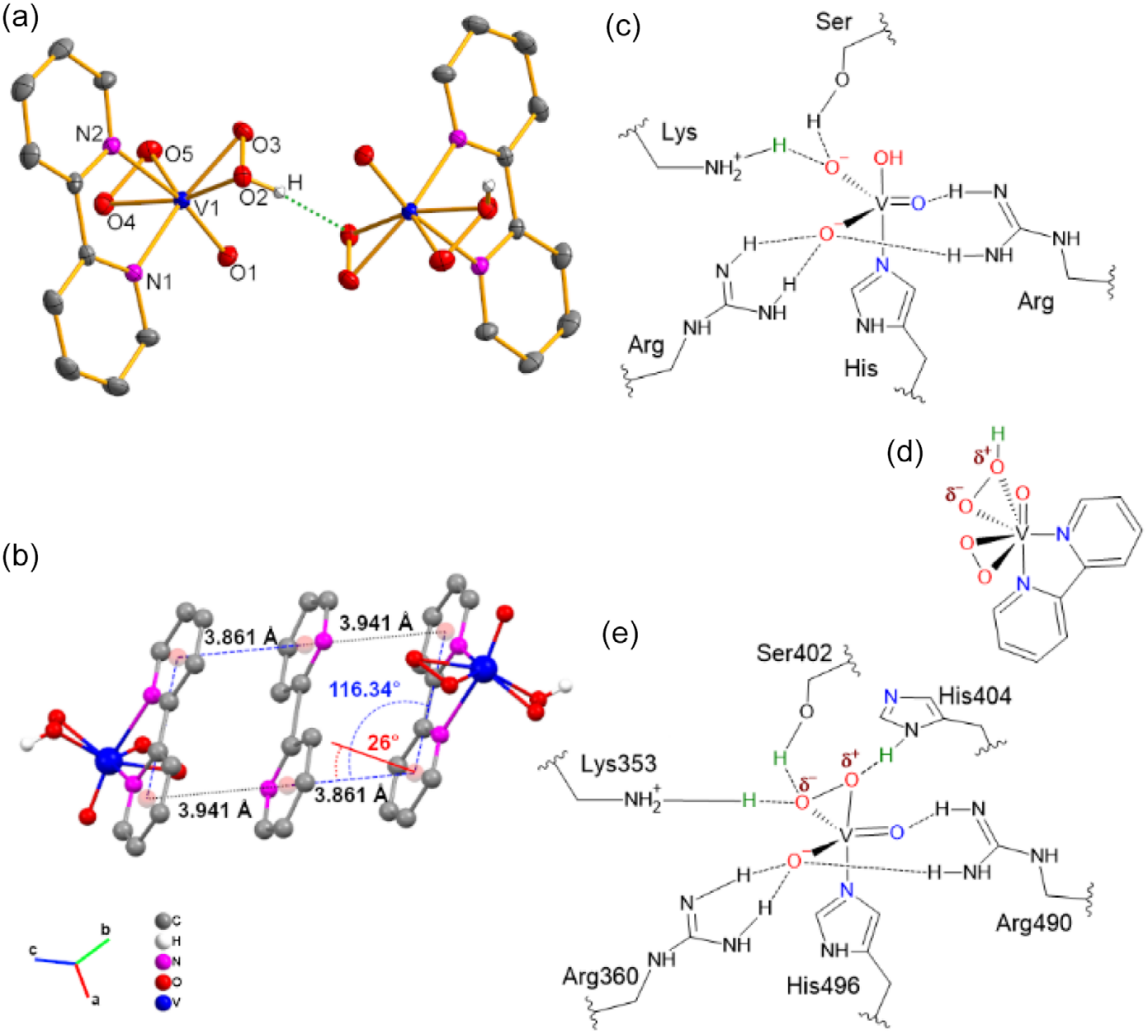
a) Molecular representation of the hydrogen bond between two molecules of 1, with thermal ellipsoids drawn at 25% probability. b) Representation of compound 1 crystalline arrangement evidencing the *π*‐stacking interactions between 2,2′‐bipyridine moieties. c) Native form of VHPO active site representation. d) V^V^O(O_2_)(HO_2_)(bpy) moiety representation. e) Active site representation described for Ci‐VCPO. Hydrogen atoms bonded to carbons were omitted for clarity.

The oxidovanadium(V) complex [V^V^O(O_2_)(HO_2_)(bpy)]·3H_2_O·0.5bpy (**1**) features a distorted pentagonal bipyramidal geometry, with a bpy ligand occupying one equatorial site alongside the *η* ^2^‐coordinated peroxide (O_2_^2−^) and hydroperoxide (HO_2_^−^) ligands (**Figure** **1**a). The asymmetric unit comprises two diperoxidovanadium moieties interconnected via strong hydrogen bonds (O···O distance ≈1.84 Å), forming a bridging network between the peroxido ligands in an alternating arrangement. The structure of **1** is analogous to that reported for the diperoxidovanadium complex containing 1,10‐phenantroline as a chelate ligand.^[^ ^30^ ^]^ In the crystalline packing of **1**, there are *π*‐stacking interactions between the bpy ligands and a crystallization 2,2′‐bipyridine, defining a chain of molecules along the c‐axis. The *π*‐stacking interaction adopts a parallel‐displaced geometry, consistent with electron‐rich aromatic rings.^[^ ^31^ ^]^ The displacement angle, defined as the deviation between the vector connecting the ring centroids and the normal vector of the reference aromatic ring,^[^ ^32^ ^]^ was measured as ca. 26° (Figure 1b). The distances observed between the crystallization 2,2′‐bipyridine centroids and the two adjacent diperoxidovanadium molecules are 3.861 and 3.941 Å, in the range of commonly observed *π*‐stacking interactions for similar ligands.^[^ ^32^ ^]^

In natural VHPOs, the vanadium‐based active site typically features a vanadium(V) center in a trigonal bipyramidal coordination geometry.^[^ ^1^ ^,^ ^33^ ^,^ ^34^ ^]^ In the enzyme's native form (Figure 1c), the vanadium is coordinated by one histidine and one hydroxyl group in the apical positions. Among the three equatorial oxygen atoms, one exhibits a slightly shorter bond length and is often described as doubly bonded to the vanadium center. The remaining two equatorial oxygens are structurally similar and engage in multiple hydrogen bonds with arginine, lysine, and serine residues. Some similarities between **1** and natural enzymes can be found in the ligands directly bonded to the vanadium center. The apical bpy mimics the histidine residue anchoring the V^V^ active site in VHPOs (e.g., His486 in An–VBPO^[^ ^34^ ^]^ and His416 in Zg‐VIPO1^[^ ^33^ ^]^), restricting the reactive part of the molecule to the *η* ^2^‐peroxido ligands (Figure 1c and d). It is noteworthy that the protonation observed for **1** resembles the hydrogen bond between Lys353 and the *η* ^2^‐peroxido ligand in Ci‐VCPO (Figure 1d and e), which polarizes the O—O bond and facilitates its reaction with the halide. Such effects were suggested by vanadium K‐edge X‐ray absorption spectroscopy studies on the Ci‐VCPO enzyme,^[^ ^35^ ^]^ and by X‐ray absorption near‐edge spectroscopy data obtained on biomimetic complexes.^[^ ^36^ ^]^ The protonated *η* ^2^‐peroxido group is a key feature of **1**, since it is well known that the catalytic activity of VHPOs and other biomimetic complexes is highly sensitive to local changes in the protonation state.^[^ ^37^ ^,^ ^38^ ^]^

**Figure 2:**
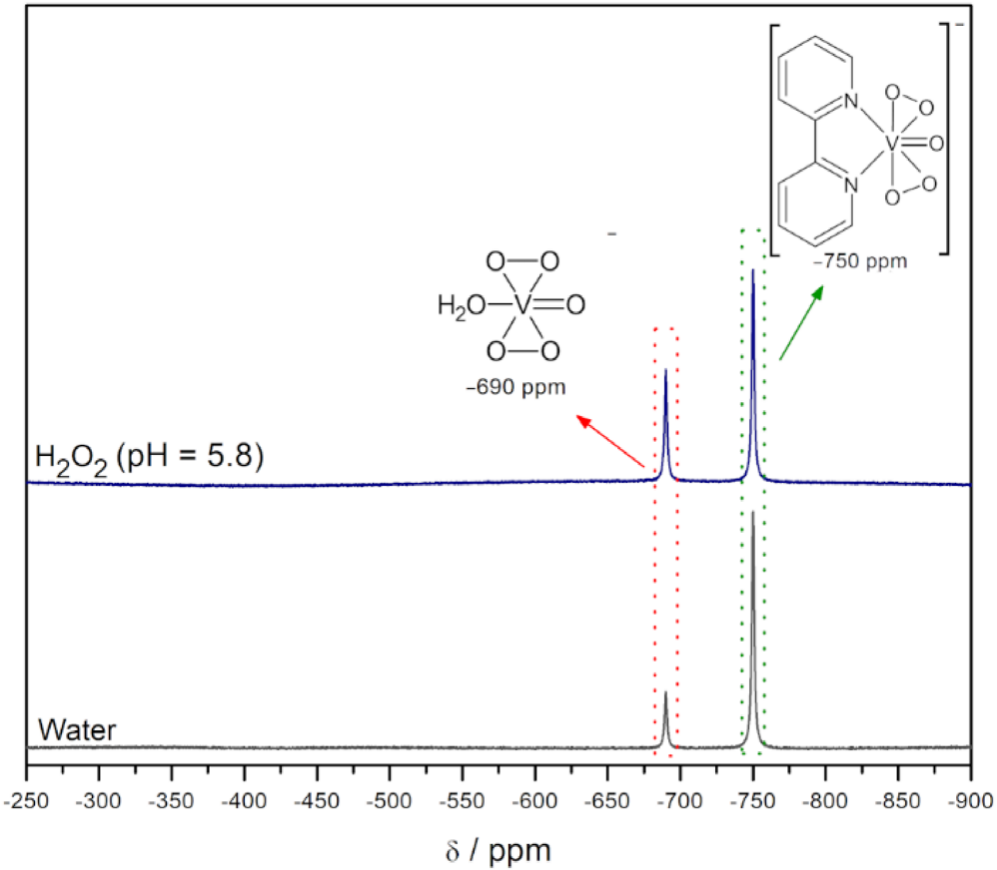
^51^V NMR spectra of **1** (0.06 mmol L^–1^) in water and phosphate buffer solution (pH 5.8, 0.24 mmol L^–1^ H_2_O_2_). The spectra were recorded for mixtures of each solution (450 µL) with the addition of D_2_O (50 µL).

In order to gain insight into the chemical species that are present in solutions of **1**, the mixtures obtained in water (0.06 mmol L^−1^) and in a medium that simulates the catalytic condition prior to the addition of substrates (phosphate buffer, pH 5.8, with 0.24 mmol L^−1^ H_2_O_2_ and 0.06 mmol L^−1^ of **1**) were analyzed by ^51^V NMR spectroscopy (**Figure** **2**). The spectra registered for both solutions presented two signals at *δ *= –690 and –750 ppm, ascribed to [VO(O_2_)_2_(bpy)]^−^ and [VO(O_2_)_2_(H_2_O)]^−^. This is in accordance with previous reports indicating that the V^V^ signal in [VO(O_2_)_2_(L)] (L = bidentate ligand) exhibits *δ *= –750 ppm and that aqueous solutions of some diperoxidovanadium complexes form one additional species, [VO(O_2_)_2_(H_2_O)]^−^, with *δ *= –690 ppm.^[^ ^39^ ^,^ ^40^ ^]^ The ^51^V NMR data indicate that [VO(O_2_)(HO_2_)(bpy)] is partially stable in solution, undergoing ligand exchange to form an equilibrium species in which the bpy ligand is replaced by water, revealing the complex's dynamic behavior under the reaction conditions.

### 2.2 DFT and Ab initio Calculations

Considering the chemical species found in solutions of **1**, the formation of HOX and V–OX species, which are responsible for the halogenation process upon reaction between the halide and the peroxidovanadium species, was explored computationally. This study was based on reaction mechanisms proposed for the VHPOs and other biomimetic catalysts.^[^ ^21^ ^,^ ^37^ ^,^ ^41^ ^,^ ^42^ ^]^ To the best of our knowledge, this is the first time in which the formation of V–OX and HOX species is modeled considering the two diperoxidovanadium species ([VO(O_2_)(HO_2_)(bpy)] and [VO(O_2_)(HO_2_)(H_2_O)]) and two halides, resulting in eight possible reaction pathways (Figure **8** and **9**).

**Scheme 1:**
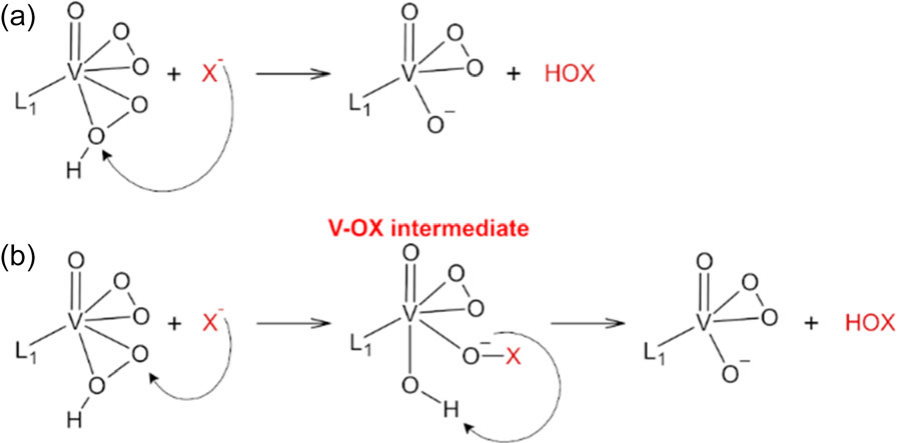
a) Representation of the nucleophilic attack of the halide on the *η* ^2^‐hydroperoxido ring protonated oxygen of [V^V^O(O_2_)(HO_2_)(L^1^)]. b) Representation of the nucleophilic attack of the halide on the *η* ^2^‐hydroperoxido ring nonprotonated oxygen of [VO(O_2_)(HO_2_)(L^1^)]. L^1 ^= bpy or water, X = Br or I.

Initially, it was proposed that the halide could attack the protonated oxygen of the OOH ligand, leading to the first reaction mechanism with iodide and bromide, **A‐I** and **A‐Br**, respectively (see **Scheme** **1**a and further discussion). This reaction path is inspired by the proposal of Mubarak et al.^[^ ^21^ ^]^ for the VHPO enzyme, in which hydrogen peroxide is bound in an end‐on mode to the oxidovanadium(V). In their model, the hydroperoxide group is attacked by the halide, which undergoes oxidation and directly forms the HOX species in a single step. Herein, it is proposed that the *η* ^2^‐hydroperoxido (side‐on) could react similarly to the end‐on hydroperoxido group, as the vanadium bond to the protonated oxygen is weak, with a V—OH bond length of ca. 2.1 Å, while the other V—O bond lengths in the VOO rings are between 1.8 and 1.9 Å. In the most accepted reaction path for VHPO enzymes, the halide can also attack the nonprotonated oxygen of the VOOH ring.^[^ ^37^ ^,^ ^41^ ^,^ ^42^ ^]^ In this work, this scenario is modeled and presented in mechanisms **B‐I** and **B‐Br** (see Scheme 1b and further discussion). After the halide approximation, the *η* ^2^‐hydroperoxido ring breaks and the halide binds to the nonprotonated oxygen, forming the V–OX intermediate, which can also undergo a prototropic shift in a second step, releasing the HOX species.

**Figure 3:**
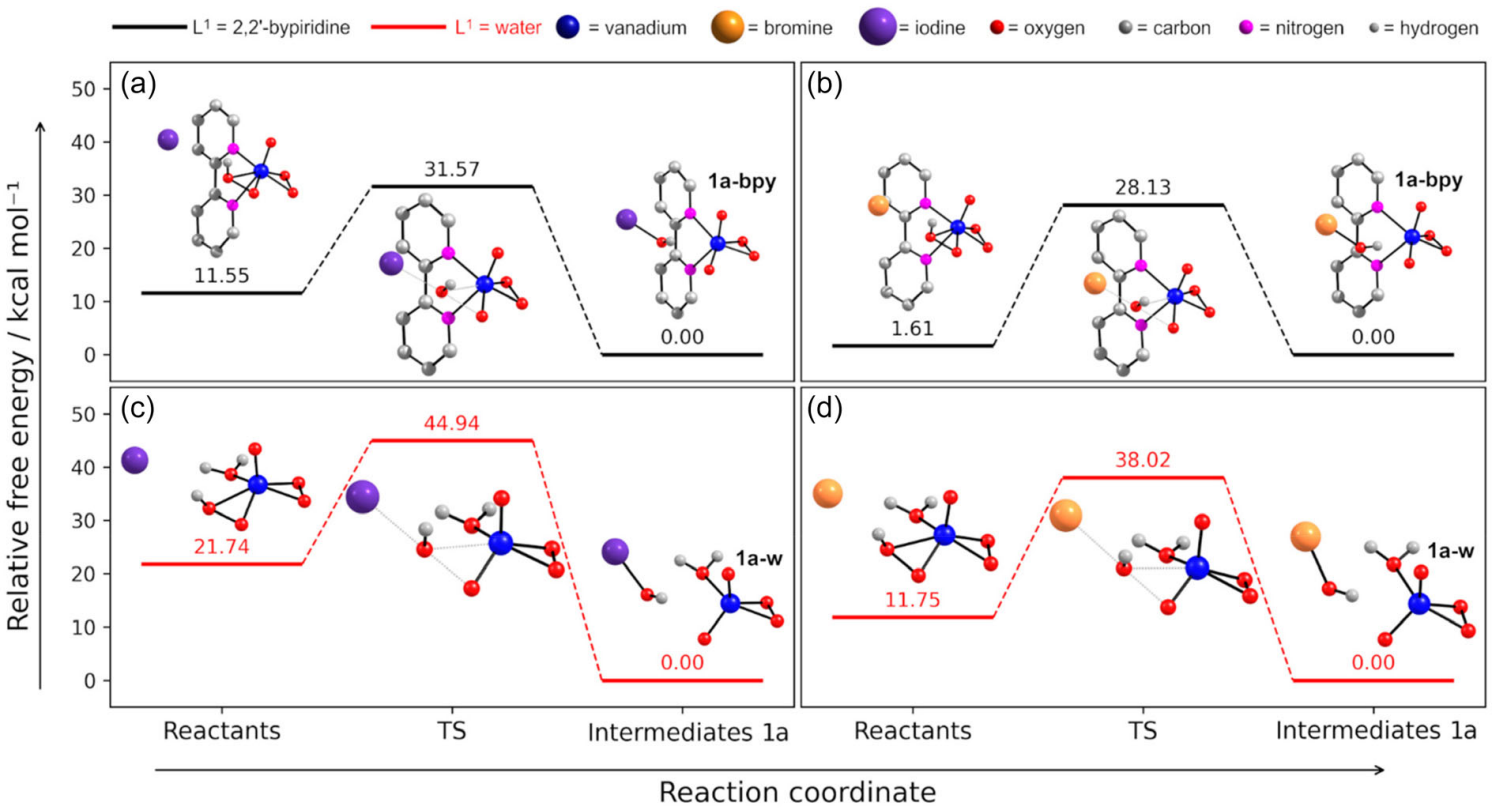
Relative free energy profile a,b) for the reaction between iodide and [VO(O_2_)(HO_2_)(bpy)] and c,d) for the reaction between bromide and [VO(O_2_)(HO_2_)(H_2_O)] (Mechanisms A‐I and A‐Br). The geometry‐optimized structures of all the molecules involved in the reaction path are represented.

In mechanism **A‐I**, a transition state (TS) was identified for the structure with L^1 ^= bpy, with an associated activation free energy, Δ*G* ^‡^, of 20.0 kcal mol^−1^, while for L^1 ^= water, the same step presented a Δ*G* ^‡^ of 23.2 kcal mol^−1^. For the reaction free energy, Δ*G*, values were –11.5 and –21.7 kcal mol^−1^ for L^1 ^= bpy or water (**Figure** **3**a and c). Although the reaction between the halide and [VO(O_2_)(HO_2_)(bpy)] showed a lower activation energy, it is remarkably less exergonic than the corresponding reaction with [VO(O_2_)(HO_2_)(H_2_O)], with a Δ*G* value roughly half as large. This mechanism leads to the formation of the intermediates **1a‐bpy** and **1a‐w**, monoperoxidovanadate compounds, releasing the hypoiodous acid.

For mechanism **A‐Br**, the activation barriers (Δ*G* ^‡^) for L^1 ^= bpy and water were 26.5 and 26.3 kcal mol^−1^, respectively, while the reaction free energies (Δ*G*) were –1.6 and –11.7 kcal mol^−1^ (Figure 3b and d). Notably, these Δ*G* values are ca. 10 kcal mol^−1^ less negative than those for the **A‐I** mechanism, and the corresponding Δ*G* ^‡^ values are ca. 5 kcal mol^−1^ higher. This energetic trend reflects the lower oxidation potential of Br^−^ compared to I^−^, which affects both the product and TS stability, resulting in higher overall energy requirements for bromide oxidation. The calculated Δ*G* ^‡^ and Δ*G* for both reactions are close to those reported by Mubarak et al. for models of the natural VCPO,^[^ ^21^ ^]^ indicating that **1** and its equilibrium counterpart are promising biomimetic models of such enzymes.

**Figure 4:**
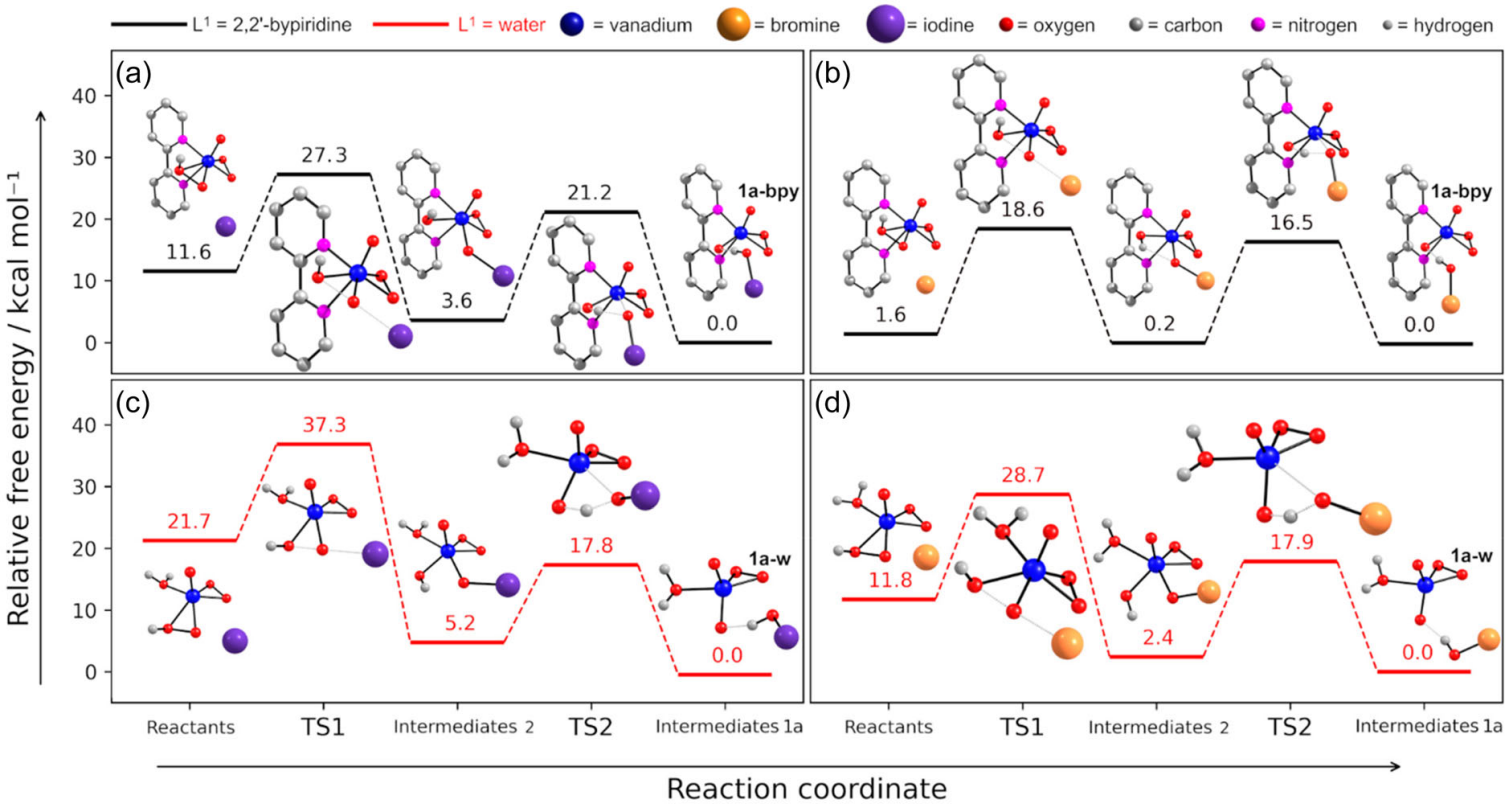
Relative free energy profile a,b) for the reaction between iodide and [VO(O_2_)(HO_2_)(bpy)] and c,d) for the reaction between bromide and [VO(O_2_)(HO_2_)(H_2_O)] (Mechanism B‐I and B‐Br). The geometry‐optimized structures of all the molecules involved in the reaction path are represented.

For mechanism **B‐I**, a first TS1 corresponding to the nucleophilic attack of the iodide to the *η* ^2^‐hydroperoxido nonprotonated oxygen was identified (**Figure** **4**a and c). For the structure with L^1 ^= bpy, this step shows an associated Δ*G* ^‡^ of 15.7 kcal mol^−1^, while for L^1 ^= water, the Δ*G* ^‡^ is 15.6 kcal mol^−1^, showing that the auxiliary ligand has minimal impact on the formation of the V–OI intermediate. Subsequently, a second TS2 was identified, in which a prototropic shift occurs within the intermediate structure, leading to the release of HOI and ultimately forming the same intermediates **1a** proposed in mechanism **A‐I**. The energy barrier for TS2 was found to be 17.6 kcal mol^−1^ for L^1 ^= bpy and 12.6 kcal mol^−1^ for L^1 ^= water (Figure 4a and c). The TS2 formed from [VO(O_2_)(HO_2_)(bpy)] seems to be less stable due to steric hindrance, as the bulkier bpy chelate occupies an additional coordination site when compared to the TS2 derived from [VO(O_2_)(HO_2_)(H_2_O)].

For the **B‐Br** mechanism, the TS1 presented Δ*G* ^‡^ values of 17.0 and 16.9 kcal mol^−1^, while TS2 showed energy barriers of 16.3 and 15.5 kcal mol^−1^, for L^1 ^= bpy or water, respectively (Figure 4b and d). Similarly to the **B‐I** mechanism, auxiliary ligands have minimal impact on the first step energy profile. However, unlike what was observed for iodide, the second reaction step showed a similar profile for both L^1^ ligands, suggesting that bromide imposes less steric hindrance on the system. The Δ*G* ^‡^ and Δ*G* for the V–OX intermediates formation are consistent with those reported for VHPO models.^[^ ^41^ ^]^ The energy profile differences between mechanisms **A** and **B** point to a slight preference for the V–OX intermediate formation over the HOX species, in good agreement with the widely accepted mechanism for VHPOs.^[^ ^1^ ^,^ ^37^ ^,^ ^41^ ^,^ ^42^ ^]^

As expected, the oxidation of bromide is less exergonic than that of iodide across all reaction pathways, consistent with the lower oxidation potential of bromide in forming hypobromous acid. Extending this trend, calculations of Δ*G* for chloride oxidation yielded positive values, confirming that the reaction with chloride is endergonic and thus nonspontaneous. Consequently, the observed Δ*G* trend follows the expected order: I^−^ < Br^−^ < Cl^−^, in agreement with the oxidation potentials of the halides.^[^ ^43^ ^]^

**Figure 5:**
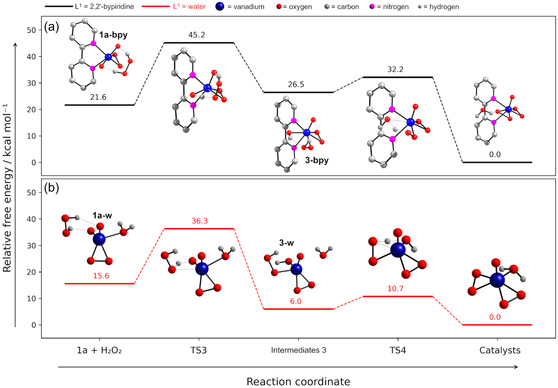
Relative free energy profile for the catalyst recovery considering both intermediates 1a. The geometry‐optimized structures of all the molecules involved in the reaction path are represented.

For catalyst recovery, two reaction mechanisms were proposed from the formation of intermediates **1a** (Figure 3 and 4), in which the respective intermediate undergoes direct reaction with hydrogen peroxide involving a two‐step process (**Figure** **5**). This proposal is also based on the reaction cycle of VHPOs and other biomimetic compounds.^[^ ^19^ ^–^ ^21^ ^]^ The first step involves a TS3 in which the end‐on coordination of the hydroperoxide ligand occurs concomitantly with a proton transfer from H_2_O_2_ to the oxidovanadium of intermediate **1a** (Figure 5a and b). This reaction step results in the formation of intermediate **3‐bpy**, which contains an end‐on hydroperoxo group for **1a‐bpy**. In contrast, for **1a‐w**, TS3 leads to the formation of intermediate **3‐w,** releasing a water molecule. This difference arises because the water ligand is a weaker Lewis base than bpy and is easily displaced. The associated Δ*G* ^‡^ values for this step are 23.6 and 20.7 kcal mol^−1^ for **1a‐bpy** and **1a‐w**, respectively, indicating that the reaction with **1a‐bpy** demands more energy than the one with **1a‐w**. The energy difference of roughly 2.9 kcal mol^−1^ might be associated with the loss of a water ligand during the hydroperoxide end‐on coordination, since this substitution process occurs through a less hindered coordination sphere when the auxiliary ligand is water.

In the second step, a prototropic shift occurs for the two reaction mechanisms, converting the end‐on peroxido into an *η* ^2^‐peroxide ligand, releasing a water molecule for **3‐bpy** and forming a coordinated water molecule for **3‐w**. Subsequently, both reactions restore the unprotonated species [VO(O_2_)_2_(L^1^)]^−^, which may undergo protonation in acidic media, restarting the catalytic cycle. It is also evident that the low barriers found for this step, ca. 5.7 and 4.7 kcal mol^−1^ for **3‐bpy** and **3‐w**, respectively, fall within the range of typical prototropic shifts. Additionally, the mechanisms proposed herein are substantially exergonic, with Δ*G* values of –32.2 and –10.7 kcal mol^−1^.

**Scheme 2:**
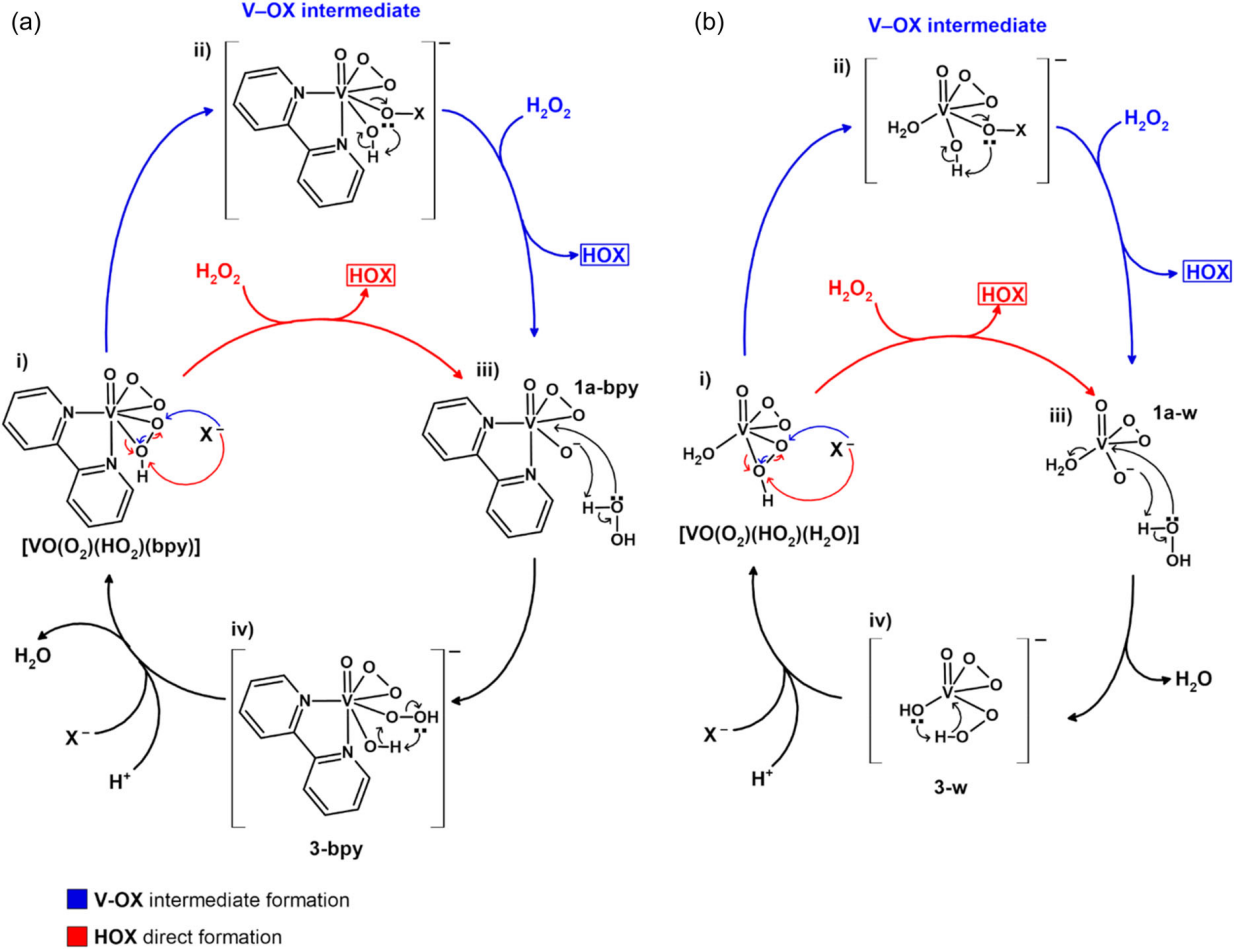
Representation of the two main reaction mechanisms proposed for the catalytic activity of a) [VO(O_2_)(HO_2_)(bpy)] and b) [VO(O_2_)(HO_2_)(H_2_O)]. The two alternative reaction pathways, including the direct formation of HOX and the formation of a V–OX intermediate, are represented with red and blue arrows, respectively.

In light of all the results, the catalyst formation and the overall reaction cycles should be considered spontaneous processes with achievable energy barriers in mild conditions, highlighting the potential of [VO(O_2_)(HO_2_)(bpy)] as a catalyst for halogenation reactions. A general schematic representation of the proposed reaction mechanisms involving the formation of V–OX intermediates, the generation of HOX, and the subsequent catalyst recovery for both [VO(O_2_)(HO_2_)(bpy)] and [VO(O_2_)(HO_2_)(H_2_O)] is presented in **Scheme** **2**a and b, respectively.

### 2.3 Catalytic Bromination Assays

**Figure 6:**
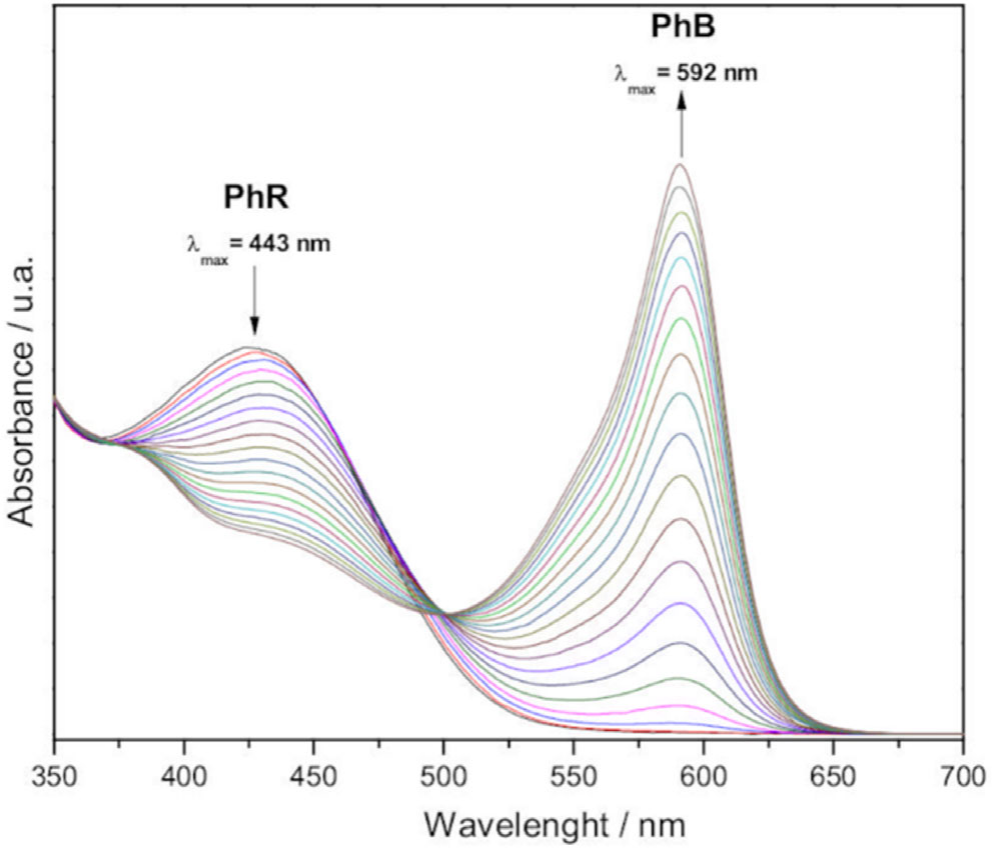
Electronic spectra registered for the PhR oxidative bromination to PhB catalyzed by 1 (1.4 × 10^−4 ^mmol L^−1^). Spectral changes were recorded every 300 s for 2 h.

The oxidative bromination of PhR to bromophenol blue (PhB) was employed as a model system to evaluate the catalytic activity of complex **1**, using a previously described methodology.^[^ ^17^ ^]^ The concentration of reactants and products was followed by electronic absorption spectroscopy. The consumption of PhR leads to a gradual decrease in the electronic absorption band at 443 nm, and the production of PhB gives rise to a new band at 592 nm showing the expected isosbestic point at 493 nm (**Figure** **6**). The blank assay, conducted without the catalyst, showed no significant spectral changes over 60 min.

**Figure 7:**
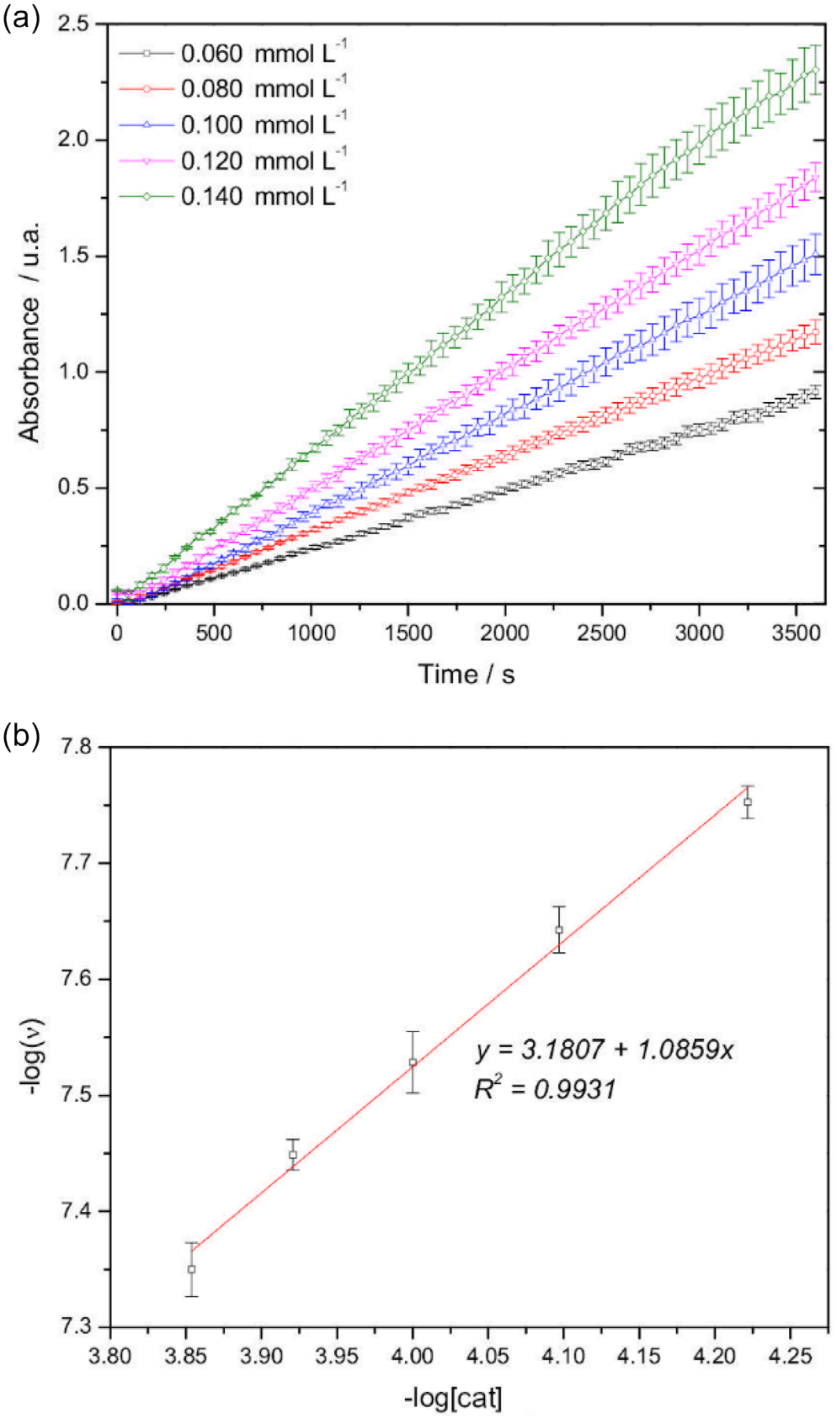
a) Absorbance plots at 592 nm as a function of time for different concentrations of complex 1. (pH = 5.8, [KBr] = 0.40 mol L^−1^, [H_2_O_2_] = 1.0 mol L^−1^, [PhR] = 0.10 mmol L^−1^, complex 1 = 6.0 × 10^−5^, 8.0 × 10^−5^, 1.0 × 10^−4^, 1.2 × 10^−4^, and 1.4 × 10^−4^ mol L^−1^). Data are presented as average values and standard deviations of triplicates (error bars: min ±0.001, max ±0.110). b) Plot of the –log(*ν*) dependence on –log[catalyst].

The catalytic effect was also studied with varying concentrations of complex **1**, from 6.0 × 10^–5^ to 1.4 × 10^–4 ^mol L^−1^, allowing the plot of the absorbance at 592 nm as a function of time (**Figure** **7**a) and the subsequent plot of log(reaction rate) as a function of the log[catalyst] (Figure 7b). The reaction order was considered to be equal to the slope of the linear fit, approaching first‐order dependence for complex **1** (1.0859), as the reaction was conducted in pseudo‐first order conditions for potassium bromide (KBr) and PhR.^[^ ^17^ ^]^ Finally, the reaction rate constant obtained of 992 ± 88 mol^2^ L^2^ min^−1^ falls in the range of values reported in the literature.^[^ ^1^ ^,^ ^17^ ^]^

**Figure 8:**
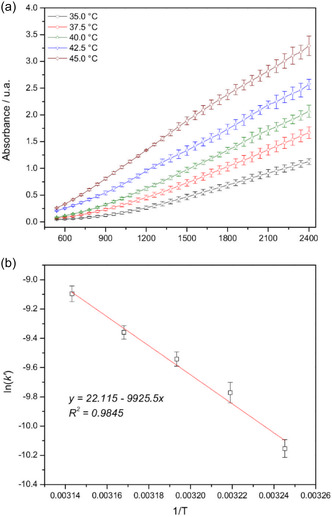
a) Absorbance plots at 592 nm as a function of time for the bromination of PhR utilizing 1 as a catalyst at different temperatures. (pH = 5.8, [KBr] = 0.40 mol L^−1^, [H_2_O_2_] = 1.0 mol L^−1^, [PhR] = 0.10 mmol L^−1^, complex 1 = 6.0 × 10^−5 ^mol L^−1^). Data are presented as average values and standard deviations of triplicates (error bars: min ±0.001, max ±0.180). b) Plot of the –ln(k′) dependence on T^–1^ obtained at 35.0, 37.5, 40.0, 42.5, and 45 ± 0.5 °C.

Further, the oxidative bromination of PhR promoted by **1** (6.0 × 10^−5 ^mol L^−1^) was explored at different temperatures (35.0, 37.5, 40.0, 42.5, and 45.0 ± 0.5 °C), producing the absorbance plot at 592 nm as a function of time (**Figure** **8**a). Subsequently, the plot of ln(k’) as a function of T^−1^ (Figure 8b) led to the reaction activation energy (E_a_) of 19.7 ± 1.5 kcal mol^−1^ from the Arrhenius equation. The E_a_ is compatible with the calculated values proposed above (ca. 20 kcal mol^−1^) and with values reported in the literature for VHPOs and biomimetic complexes (15–20 kcal mol^−1^).^[^ ^15^ ^,^ ^20^ ^,^ ^21^ ^]^

### 2.4 8–hydroxyquinoline Halogenation

**Figure 9:**
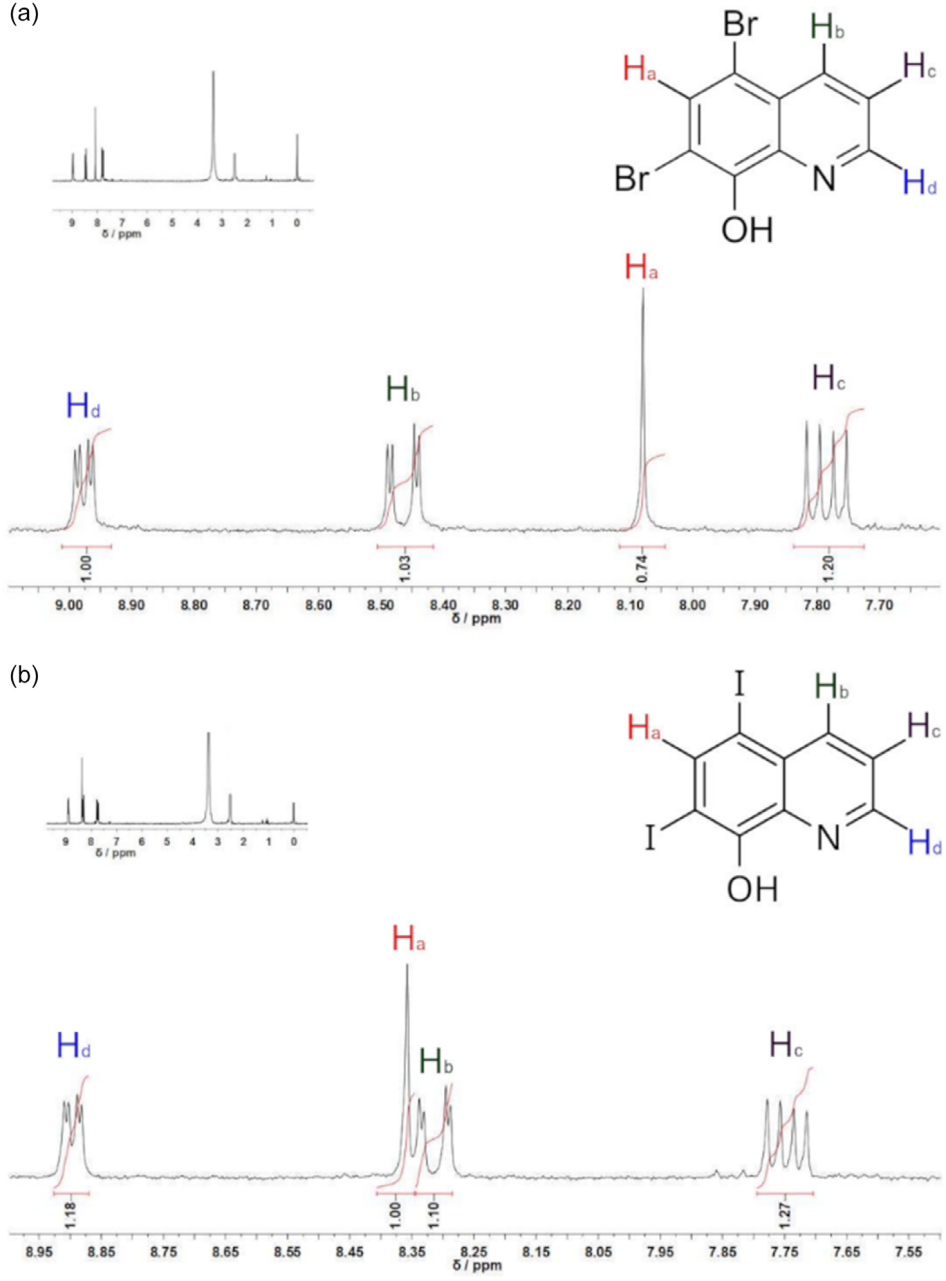
a) ^1^H NMR spectrum (400 MHz, dmso‐d_6_) of 5,7‐dibromoquinolin‐8‐ol obtained using complex 1 as a catalyst for oxidative bromination. b) ^1^H NMR spectrum (400 MHz, dmso‐d_6_) of 5,7‐diiodoquinolin‐8‐ol obtained using complex 1 as a catalyst for oxidative bromination. The expanded spectra are shown as inserts in each graph.

The potential of **1** as a catalyst for the halogenation of 8‐hydroxyquinoline was also considered, as its bromination and iodination products are of commercial relevance due to their pharmaceutical use.^[^ ^27^ ^,^ ^44^ ^]^ In this context, the halogenation reactions of this substrate were carried out under mild conditions for 72 h. The dihalogenated products, 5,7‐dibromoquinolin‐8‐ol and 5,7‐diiodoquinolin‐8‐ol, were obtained in 73% and 86% yields, respectively. ^1^H NMR (**Figure** **9**) showed only the signals expected for the desired products, attesting to the purity of the isolated solids. The signal observed for the H_a_ atom, adjacent to two halides, is less shielded for the product containing iodine than bromine. Additionally, the ^13^C NMR analysis shows the signals expected for the carbons; however, the chemical shifts are impacted by the halogen atoms (Figure S4, Supporting Information). Notably, the reaction proceeded at only 30.0 ºC, highlighting the feasibility of halogenation under mild conditions when using complex **1** as a catalyst. As far as we know, the present work is the first report on both bromination and iodination of 8‐hydroxyquinoline employing a vanadium biomimetic complex.

It is also worth noting that analogous oxidative halogenation reactions have been reported for a variety of substrates, including thymol and several aromatic molecules, using different transition metal oxido‐ and peroxido‐ complexes, including molybdenum(VI) and tungsten(VI).^[^ ^45^, ^46^, ^47^ ^–^ ^48^ ^]^ This parallel suggests that the insights gained in this work could be extrapolated to other catalytic systems, thereby contributing to future investigations into sustainable and efficient green halogenation methodologies.

## 3 Conclusion

This study explores the bipyridine‐coordinated peroxidovanadium [V^V^O(O_2_)(HO_2_)(bpy)]·3H_2_O·0.5bpy (**1**) as a biomimetic catalyst for oxidative halogenation of organic substrates, approximating enzymatic and synthetic systems. Structural and mechanistic analyses reveal that complex **1** mimics key features of VHPOs, including: 1) a protonated *η* ^2^‐peroxide ligand featuring a reactive, polarized O—O bond capable of halide oxidation and 2) a flexible coordination sphere that stabilizes V–OX intermediates and facilitates direct HOX formation.

Computational studies were based on the presence of two chemical species, the [V^V^O(O_2_)(HO_2_)(bpy)] and its equilibrium counterpart [V^V^O(O_2_)(HO_2_)(H_2_O)], in buffered solutions of **1** (pH = 5.8), considering the most common halides oxidized by VHPOs. For each catalytic species, two main mechanisms were proposed, the first involving direct formation of HOX and the second forming the V–OX intermediate before the prototropic shift releases the HOX species.

The HOX direct formation presents similar energy barriers for both iodide and bromide oxidation steps, while their Δ*G* values differ by ca. 10 kcal mol^−1^. This difference is related to the oxidation potential of both halides, where iodide is more easily oxidized than bromide, reflecting the higher stability of HOI compared to HOBr. The same trend is observed for the V–OX intermediate pathway, with Δ*G* ^‡^ values lower than those observed for the direct HOX formation, indicating a slight preference for the second reaction mechanism. The energy barriers for the catalyst regeneration (ca. 20 kcal mol^−1^) fall in the range of energies easily accessible in near ambient temperature conditions,^[^ ^49^ ^]^ underscoring the potential of vanadium catalysts for the halogenation of organic substrates in mild conditions.

Experimentally, **1** is an efficient catalyst for the PhR bromination, with a rate constant of 992 mol^2^ L^2^ min^−1^ and presenting a Δ*G* ^‡^ of c.a. 20 kcal mol^−1^, in good agreement with the DFT/DLPNO‐CCSD(T) calculated values. Considering the pharmaceutical industry's need for greener halogenation processes, **1** was employed for the halogenation of 8‐hydroxyquinoline, due to the antimicrobial properties of its products. For both reactions, the dihalogenated compounds were obtained as solids in high yield, without additional purification steps. These findings help advance the rational design of sustainable halogenation processes for organic substrates by employing vanadium‐based catalysts for future pharmaceutical and agrochemical industries.

## 4 Computational Details

All theoretical calculations utilized the ORCA 6.0.1 software package.^[^ ^50^ ^]^ Intermediates and TSs underwent optimization via DFT with the DKH‐def2‐TZVP^[^ ^51^ ^]^ basis set, employing the *ω*B97X functional^[^ ^52^ ^]^ with D3 dispersion correction^[^ ^53^ ^]^ for all atoms. To account for relativistic effects, the Douglas–Kroll–Hess second‐order approximation^[^ ^54^ ^]^ was applied. Computations were performed with the RIJCOSX approximation,^[^ ^55^ ^]^ ensuring efficiency without sacrificing accuracy, in conjunction with automatically generated auxiliary basis sets.^[^ ^56^ ^]^ TSs were characterized by a single imaginary vibrational frequency along the reaction coordinate, as confirmed through harmonic frequency analysis. Solvent effects were modeled using the conductor‐like polarizable continuum model,^[^ ^57^ ^,^ ^58^ ^]^ with water as the solvent. Final electronic energies stemmed from single‐point calculations at the DLPNO‐CCSD(T),^[^ ^59^ ^,^ ^60^ ^]^ theory level, using the DKH‐def2‐TZVPP basis set^[^ ^51^ ^]^ on the DFT‐optimized geometries. Total free energies were obtained by combining DFT thermal corrections with CCSD(T) electronic energies. Molecular graphics were generated using Chemcraft 1.8.^[^ ^61^ ^]^

## Supporting Information

Experimental details (materials, physical measurements, synthesis, characterization, and catalytic procedures) are available in the Supporting Information.^[62–67]^

## Conflict of Interest

The authors declare no conflict of interest.

## Supporting information

Supplementary Material

## Data Availability

The data that support the findings of this study are available in the supplementary material of this article.
