# Supplementary material for: Mechanistic Insights and Catalytic Efficiency of a 2,2′‐Bipyridine–Coordinated Peroxidovanadium Complex as a Haloperoxidase Biomimetic
Source: Chempluschem. 2025 Sep 1;90(11):e202500444. doi: 10.1002/cplu.202500444 (PMC12605662; doi:10.1002/cplu.202500444)
Supplement: Supplementary file 1 — Supplementary Material [file CPLU-90-e202500444-s001.pdf]

Supplementary material: Mechanistic Insights and Catalytic Efficiency of a 2,2’-Bipyridine–Coordinated Peroxidovanadium Complex as a Haloperoxidase Biomimetic

Lucas G. Fachini[a], Enzo V. S. Elisandro[a], Gabriel B. Baptistella[a], Rúbia C. R. Bottini[b], Matteo Briganti[c], Giovana G. Nunes[a], Eduardo L. de Sá[a]*

[a] L. G. Fachini, E. V. S. Elisandro, Dr. G. B. Baptistella, Prof. G. G. Nunes, Prof. E. L. de Sá
Departamento de Química
Universidade Federal do Paraná
Centro Politécnico, Jardim das Américas, 81530-900, Curitiba, PR, Brazil
E-mail:edulsa@ufpr.br

[b] Dr. R. C. R. Bottini
Departamento de Química e Biologia
Universidade Tecnológica Federal do Paraná
Rua Deputado Heitor de Alencar Furtado, Ecoville, 81280-340, Curitiba, PR, Brazil

[c] Prof. M. Briganti
Dipartimento di Chimica Ugo Schiff and INSTM RU
Università degli Studi di Firenze
Via della Lastruccia 3–13, 50019 Sesto Fiorentino–FI, Italy

Experimental details

Materials

The reactants: 97% $VOSO_4 \cdot nH_2O$ (n =4-5), 98% $NaVO_3$, 99% citric acid or 99% R,S-malic acid and 99% 2, 2’–bipyridine were supplied by Sigma–Aldrich. 30% NH_4OH, 37% HCl, and 35% H_2O_2 were supplied by Êxodo Científica. For bromination assays it was also employed KBr, KI, NaH_2PO_4, Na_2HPO_4 and Phenol red from Sigma–Aldrich with purity above 98%. The synthesis was conducted using deionized water (Permution®, type 3, conductivity ≤ 3.5 μS cm^{-1} at 25 ºC) and the NMR analyses were carried out employing 99.8% deuterium oxide and 99.9% dmso–d6 as received from Sigma–Aldrich.

Physical Measurements

Carbon, hydrogen, and nitrogen contents were determined through combustion in a Perkin Elmer 2400 Series II Elemental Analyzer. Vanadium(V) quantification followed a titrimetric method described in the literature[1] and thermogravimetric assays. The X–ray powder diffraction (PXRD) pattern was obtained from a Shimadzu XRD–600 diffractometer, operating at 40 kV and 40 mA with a Cu–target tube (Cu–Kα, λ = 1.5418 Å) over a 2θ range of 3–50°. The simulated powder X–ray diffractogram of complex 1 was calculated from the single–crystal crystallographic information file (CIF) using Mercury 4.0 software[2]. Electronic absorption spectra were measured with a PerkinElmer Lambda 650 UV–Vis–NIR spectrophotometer,

coupled to a Peltier temperature control system (The Research Peltier® System, PTP 1+1, PerkinElmer). Infrared (IR) spectra, collected from KBr pellets, used a Vertex 70v FT–IR spectrophotometer with a resolution of 2 cm^{-1} in the 400–4000 cm^{-1} range. The ^{51}V NMR spectra, recorded at 295 K, utilized a Bruker 400 MHz Avance III spectrometer (9.4 T) equipped with a 5 mm multinuclear probe. Sample preparation involved dissolving compounds in aqueous solutions (450 µL) with the addition of 50 µL of D_2O. Spectra acquisition included 2048 scans, a 90° pulse, a 0.100 s recycling delay, a 0.16 s acquisition time, and a spectral width of 714 ppm (−900 to −186 ppm). $VOCl_3$ (neat, capillary, 0.00 ppm) was used as reference, with detection at 105.2 MHz. Spectral intensities underwent normalization to the reference signal in each experiment. ^1H and ^{13}C NMR spectra were collected dissolving the product in the deuterated solvent (DMSO-d_6 or CD_3Cl), utilizing the same spectrometer for ^1H and using a Bruker 600 MHz spectrometer (14.1 T) for the ^{13}C analysis.

Synthesis of [$V^VO(O_2)(HO_2)(bpy)$]·$3H_2O$·0.5bpy (1)

For the synthesis of 1, 0.50 mmol of $NaVO_3$ (45.0 mg) was dissolved in a 2:1 (v/v) mixture of water and ethanol (50 mL), producing a yellow solution. To this solution, 1.20 mmol of hydrogen peroxide (70 µL) was added, and the mixture was magnetically stirred for 20 min. The solution was then acidified with diluted HCl to pH 3.0, followed by the addition of 0.50 mmol of 2,2'–bipyridine (78.1 mg). The resultant clear orange solution was stirred for 60 min, left to crystallize for 7 days at 4 °C and the yellow solid was filtered off, rinsed with 5 mL of cold water and dried in air, yielding 39%. Elemental analysis: calculated for $C_{15}H_{20}N_3O_8V$: V, 12.09%; C, 42.77%; H, 4.78%; N, 9.97%; Found: V, 12.37%; C, 42.58%; H, 3.40%; N, 9.78%. Complex 1 was additionally characterized by FTIR (Fig. S3 and Table S2) and UV–VIS spectroscopies (Fig. S2) and by diffractometric techniques, SC-XRD (Table S1) and PXRD (Fig. S1).

Single–crystal X–ray data collection

A yellow prismatic crystal of complex 1 was selected to be analyzed at 100(2) K on a Bruker D8 Venture diffractometer equipped with a Photon 100 CMOS detector, using Mo–Kα radiation (0.71073 Å) and graphite monochromator. Data were processed using the APEX 3 program[3]. The structure was determined by dual methods in SHELXT software[4] and refined by full–matrix least–squares methods, on F^2's, in SHELXL[5]. Scattering factors for neutral atoms were taken from the literature[6]. The computer programs used for refining were run through WinGX[7]. Details on data collection and structure refinement for 1 are presented in Table S1.

Catalytic Bromination measurements

The catalytic bromination of phenol red (PhR), producing bromophenol blue (PhB) was conducted using 1 as the catalyst, according to the method already described[8]. The reaction was carried at 30.0, 35.0, 37.5, 40.0, 42.5 and 45.0 ± 0.5 °C in buffered medium (NaH_2PO_4/Na_2HPO_4, pH = 5.8) with phenol red (0.10 mmol L^{-1}), KBr (0.40 mmol L^{-1}) and H_2O_2 (1.0 mmol L^{-1}) to which it was added a aqueous solution of the catalyst to give the final desired concentration. The reaction was monitored through recording of UV/Vis spectral changes in 60 s intervals. Every experiment was conducted independently three times.

The reaction rate constant was determined following previous works[8], where the differential form of Beer–Lambert's law is employed, dA/dt = ε b (d[PhB]/dt), considering A as the absorbance of the sample at 592 nm, b being the light pathlength across the sample cell (1.0 cm) and ε being the molar absorption coefficient for phenol red at 592 nm (14500 L mol^{-1} cm^{-1}). The reaction rate (v) law is expressed in the Eq. 1, where α, β and γ are the reaction orders for the catalyst (cat), bromide and PhR, respectively.

$$\mathrm{v} = \frac{\mathrm{d[PhB]}}{\mathrm{dt}} = \frac{1}{\varepsilon\,\mathrm{b}}\frac{\mathrm{d[A]}}{\mathrm{dt}} = \mathrm{k[cat]}^{\alpha}[\mathrm{Br}^-]^{\beta}[\mathrm{PhR}]^{\gamma} \qquad (1)$$

Assuming pseudo–first–order conditions the plot of absorbance in function of time is a straight line with slope equals to vεb. A plot of –log(v) in function of –log([cat]) makes it possible to obtain α as the slope and also k from the intercept.

Furthermore, to estimate the reaction activation energy, the catalytic bromination can be performed fixing the catalyst concentration and varying the temperature. This method is based on the differential Beer–Lambert equation, where k' is defined as $k[Br^-]^{\beta}[PhR]^{\gamma}$, and α is set to 1, allowing the plot of log v = log k' + α log[cat], with log k' as the intercept. Finally, the obtained ln(k') values are plotted against T^{-1} as suggested by the Arrhenius equation, in which E_a is determined by the curve slope (Equation 2).

$$\mathrm{k}' = \mathrm{A}e^{-\frac{\mathrm{E_a}}{\mathrm{R}}\frac{1}{\mathrm{T}}} \Rightarrow \ln(\mathrm{k}') = \ln\mathrm{A} - \frac{\mathrm{E_a}}{\mathrm{R}}\frac{1}{\mathrm{T}} \qquad (2)$$

Halogenation of 8–hydroxyquinoline

The 8–hydroxyquinoline (0.060 mmol, 8.8 mg) was dissolved in 1:1 mixture of ethanol and a NaH_2PO_4/Na_2HPO_4 buffer solution (50 mmol L^{-1}, 25 mL, pH 5.8), followed by the addition of 0.24 mmol H_2O_2 (15 µL), 0.0015 mmol of complex 1 (1.26 mg) and 0.24 mmol of either KBr (0.029 g) or KI (0.040 g), depending on the halogenation process. The mixture afforded limpid solutions which were maintained under constant magnetic stirring for 72 h. The products were formed as suspended solids that were filtered off, rinsed with cold water, and dried under vacuum in a desiccator, resulting in 13.96 mg of 5,7–dibromoquinolin–8–ol (73% yield) and 20.47 mg of 5,7–diiodoquinolin–8–ol (86% yield). The products were characterized by ^{1}H NMR analysis.

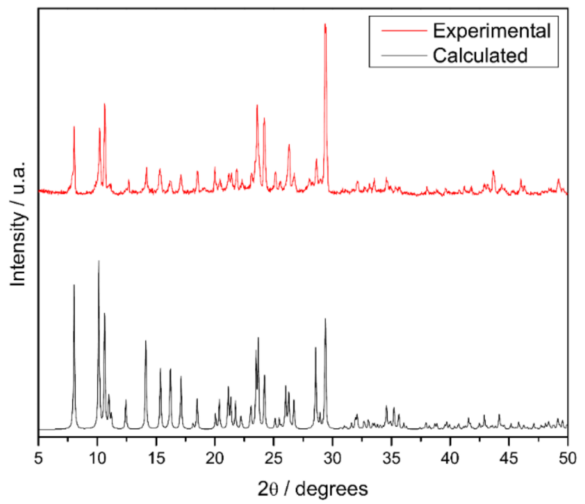

Figure S1. Powder X-ray diffraction pattern for $[V^{V}O(O_2)(HO_2)(bpy)]\cdot3H_2O\cdot0.5bpy$ obtained experimentally and calculated from Single Crystal X-ray diffraction data.

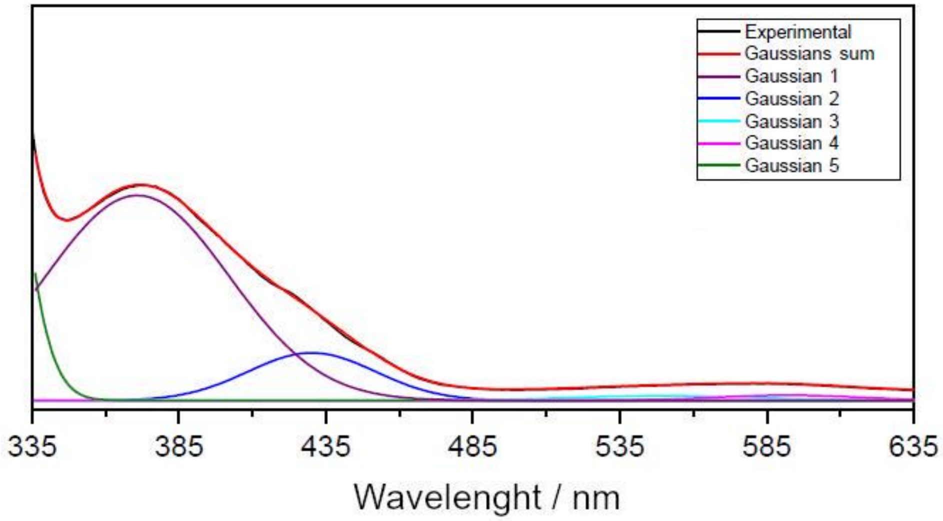

Figure S2. $[V^{V}O(O_2)(HO_2)(bpy)]\cdot3H_2O\cdot0.5bpy$ UV/Vis spectrum in water solution at a concentration of 1.0 mmol L^{-1} with addition of 0.01 mmol L^{-1} of H_2O_2 and its decomposition into gaussian sub-bands. The bands with λ_{max} at 371 and 432 nm are assigned to LMCT electronic transitions from the π orbitals of the η^2-peroxido ligands to the vanadium center, characteristic of diperoxido vanadium complexes.

Table S1. Crystal data and structure refinement for $[V^{V}O(O_2)(HO_2)(bpy)]\cdot 3H_2O\cdot 0.5bpy$.

Identification code	LGF0124
Elemental formula	$(C_{10}H_9N_2O_5V)$, $0.5(C_{10}H_8N_2)$, $3(H_2O)$
Formula weight	421.28
Crystal system, space group	Triclinic, P-1
Unit cell dimensions	a = 9.1120(6) Å α = 100.855(2) °<br>b = 9.4554(5) Å β = 111.473(2) °<br>c = 12.1473(8) Å γ = 103.988(2) °
Volume	899.58(10) $Å^3$
Z, calculated density	2, 1552 Mg m^{-3}
F(000)	434
Absorption coefficient	0.601 mm^{-1}
Temperature	273(2) K
Wavelength	0.71073 Å
Crystal colour, shape	Yellow diamond
Crystal size	0.665 x 0.535 x 0.409 mm
Crystal mounting:	On a glass fiber, in oil
On the diffractometer:	
Theta range for data collection	2.513 to 33.312 °
Limiting indices	-17<=h<=17, -11<=k<=11, -26<=l<=26
Completeness to theta = 25.242	99.0 %
Absorption correction	Semi-empirical from equivalents
Max. and min. transmission	0.785 and 0.690
Reflections collected / unique	96601 / 6954 [R(int) = 0.028]
No. of 'observed' reflections ($I > 2\sigma I$)	5837
Structure determined by:	dual methods, in SHELXS
Refinement:	Full-matrix least-squares on F2, in SHELXL
Data / restraints / parameters	6878 / 9 / 293
Goodness-of-fit on F^2	1.070
Final R indices ('observed' data)	R1 = 0.046, wR2 = 0.123
Final R indices (all data)	R1 = 0.058, wR2 = 0.136
Reflections weighted:	$w=[\sigma^2(Fo^2)+(0.0637*P)^2+0.5573P]^{-1}$ where $P=(Fo^2+2Fc^2)/3$
Extinction coefficient	n/a
Largest diff. peak and hole	0.91 and -0.48 e. Å-3
Location of largest difference peak	n

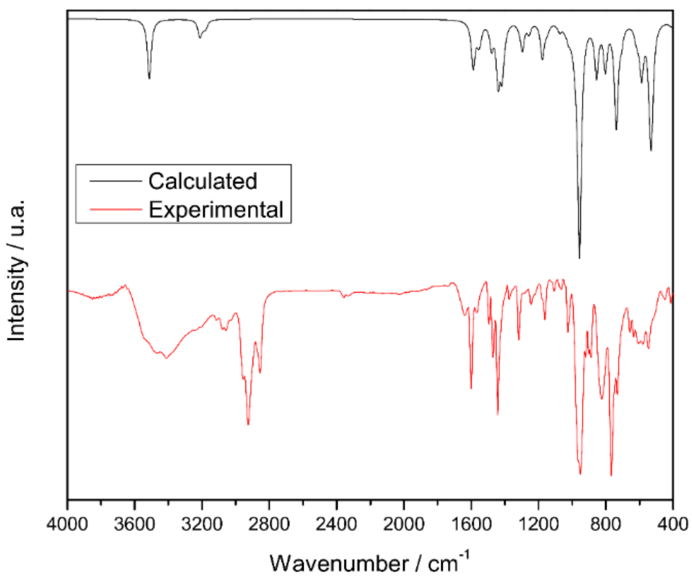

Figure S3. Infrared spectra of $[V^{V}O(O_2)(HO_2)(bpy)]\cdot 3H_2O\cdot 0.5bpy$ recorded from samples prepared in KBr pellets at a concentration of 1 % (m/m).

Table S2. Tentative assignments for IR bands registered for $[V^{V}O(O_2)(HO_2)(bpy)]\cdot 3H_2O\cdot 0.5bpy$.

Tentative assignment	Experimental
ν(O-H)	Over 3100
$\delta(C\text{-}H)_{bipy}$	1317
$\nu(C\text{-}C)_{bipy}$	1442, 1599
$\delta(C\text{-}H)_{bipy}$	768
ν(V=O)	953
ν(V-O)	546
ν(O-O)	826 and 887

ν = stretching vibrational mode, δ = angular deformation vibrational mode, bipy = 2,2'-bipyridine

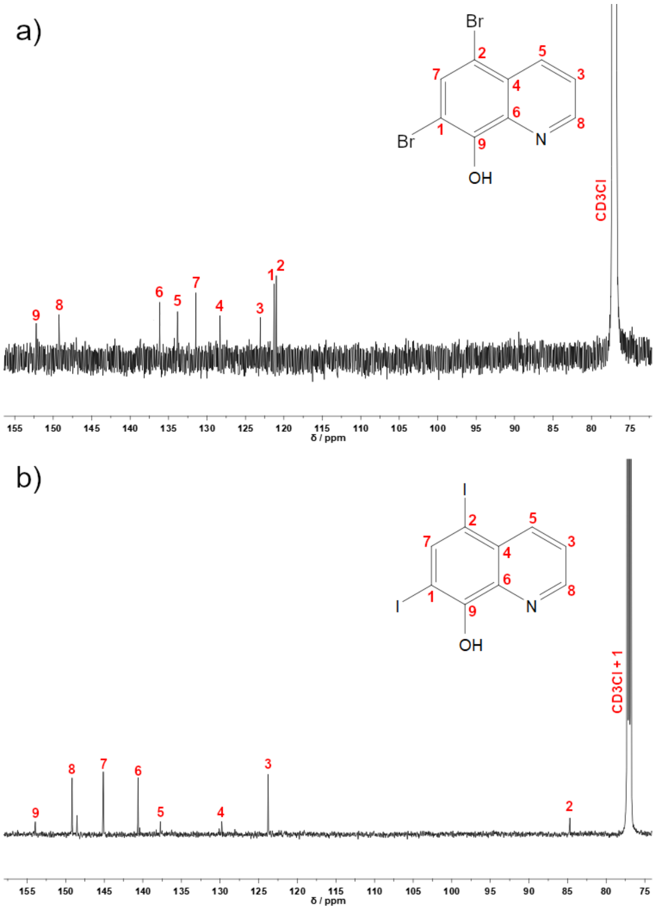

Figure S4. a) ^{13}C NMR spectrum (600 MHz, CD_3Cl) of 5,7-diiodoquinolin-8-ol obtained using complex 1 as a catalyst for oxidative bromination. b) ^{13}C NMR spectrum (600 MHz, CD_3Cl) of 5,7-dibromoquinolin-8-ol obtained using complex 1 as a catalyst for oxidative bromination.

REFERENCES

[1] D. Wang, H. H. Kung, M. A. Barteau, *Appl. Catal. A Gen.* **2000**, *201*, 203–213.

[2] C. F. Macrae, I. Sovago, S. J. Cottrell, P. T. A. Galek, P. McCabe, E. Pidcock, M. Platings, G. P. Shields, J. S. Stevens, M. Towler, *J. Appl. Crystallogr.* **2020**, *53*, 226–235.

[3] A. X. S. Bruker, *APEX3, SAINT and SADABS; Bruker AXS Inc.: Madison, WI, USA* 2016.

[4] G. M. Sheldrick, *Acta Crystallogr. A* **2015**, *71*, 3–8.

[5] G. M. Sheldrick, *Acta Crystallogr. C* **2015**, *71*, 3–8.

[6] D. C. Creagh, W. J. McAuley, *Vol C,(AJC Wilson, ed.), Kluwer Academic Publishers, Boston, Table* 1992, *4*, 200–206.

[7] L. J. Farrugia, *J. Appl. Crystallogr.* **2012**, *45*, 849–854.

[8] R. C. R. Bottini, L. G. Fachini, G. B. Baptistella, D. Stinghen, F. S. Santana, M. Briganti, R. R. Ribeiro, J. F. Soares, E. L. Sá, G. G. Nunes, *Inorg. Chim. Acta* **2022**, *537*, 120947.

Cartesian coordinates optimized at the ωB97X-D3/ DKH–def2–TZVP level in water for all stationary points discussed in the main text. The structures are labeled according to their role in the reaction mechanism, except for the starting reactants and simple molecules, which are denoted by their chemical formulae:

[VO(O$_2$)(HO$_2$)(bpy)]

Element and coordinates (Å, X, Y, Z)			
V	-2.053341000	0.332765000	0.742920000
C	-1.369919000	-2.050646000	-1.017062000
C	-0.667239000	-2.868769000	-1.875499000
C	0.617095000	-2.507386000	-2.225697000
C	1.150356000	-1.345796000	-1.705665000
C	0.393619000	-0.575689000	-0.850621000
C	0.887686000	0.671504000	-0.248236000
C	2.139935000	1.193129000	-0.493640000
C	2.502352000	2.373705000	0.124442000
C	1.606346000	2.993351000	0.972563000
C	0.373987000	2.402443000	1.171040000
N	-0.855906000	-0.928569000	-0.516225000
N	0.027256000	1.271650000	0.571766000
O	-1.892317000	0.837137000	2.553074000
H	-2.380220000	-2.284116000	-0.709823000
H	-1.134150000	-3.768447000	-2.251795000
H	1.199376000	-3.124669000	-2.897781000
H	2.155643000	-1.051312000	-1.968541000
H	2.834898000	0.699024000	-1.156988000
H	3.478677000	2.806237000	-0.053426000
H	1.845095000	3.917697000	1.480732000
H	-0.367563000	2.835000000	1.831241000
O	-3.179526000	-0.768788000	0.840022000
O	-1.074636000	-0.325684000	2.474647000
O	-2.865428000	1.934469000	0.410014000
O	-2.444570000	1.287447000	-0.771978000
H	-1.606773000	-1.021436000	2.893322000

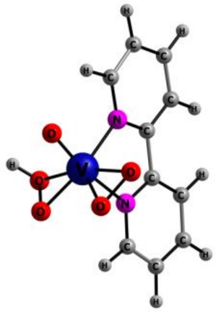

Electronic energy: -1821.86945294 Eh

[VO(O$_2$)(HO$_2$)(H$_2$O)]

Element and coordinates (Å, X, Y, Z)			
V	-2.054524000	0.585701000	0.766041000
O	-2.308587000	0.802281000	2.593682000
O	-2.585415000	-0.816181000	0.339026000
O	-1.058471000	0.127657000	2.493546000
O	-3.399791000	1.786188000	0.491879000
O	-2.434881000	1.795096000	-0.537872000
H	-1.231711000	-0.780580000	2.796060000
O	-0.163206000	0.732453000	0.013416000
H	-0.034444000	1.071627000	-0.880283000
H	0.480149000	0.028181000	0.159763000

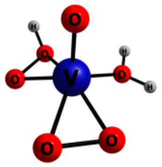

Electronic energy: -1402.80838303 Eh

**A-I (bpy) TS**				**A-Br (bpy) TS**			
Element and coordinates (Å, X, Y, Z)				Element and coordinates (Å, X, Y, Z)			
V	-2.099961000	0.312237000	0.848143000	V	-2.081997000	0.263561000	0.837903000
C	-1.358942000	-2.062170000	-0.994006000	C	-1.323247000	-2.059615000	-1.051796000
C	-0.625055000	-2.875630000	-1.830323000	C	-0.611274000	-2.852144000	-1.928238000
C	0.637418000	-2.473888000	-2.210396000	C	0.667792000	-2.466802000	-2.272579000
C	1.122771000	-1.285106000	-1.717984000	C	1.180480000	-1.305715000	-1.732848000
C	0.351105000	-0.539380000	-0.853451000	C	0.408136000	-0.563797000	-0.864284000
C	0.858433000	0.693776000	-0.235359000	C	0.883288000	0.682631000	-0.243210000
C	2.128916000	1.165652000	-0.490810000	C	2.129301000	1.225926000	-0.481616000
C	2.533337000	2.338473000	0.109288000	C	2.475579000	2.401957000	0.153068000
C	1.662998000	2.995712000	0.956939000	C	1.569425000	2.996251000	1.009133000
C	0.421171000	2.430259000	1.190276000	C	0.346718000	2.381715000	1.199214000
N	-0.884260000	-0.913734000	-0.514682000	N	-0.830040000	-0.940438000	-0.532677000
N	0.030468000	1.307412000	0.602852000	N	0.014645000	1.257261000	0.582533000
O	-1.925320000	0.810227000	2.519592000	O	-1.917136000	0.802471000	2.491941000
H	-2.355544000	-2.320425000	-0.668558000	H	-2.328485000	-2.311934000	-0.741545000
H	-1.047724000	-3.808808000	-2.173875000	H	-1.064840000	-3.752095000	-2.320371000
H	1.242036000	-3.082969000	-2.867783000	H	1.260163000	-3.064485000	-2.953807000
H	2.108152000	-0.951849000	-1.997463000	H	2.180519000	-0.989029000	-1.990924000
H	2.805110000	0.628805000	-1.135994000	H	2.832463000	0.750130000	-1.149906000
H	3.523111000	2.736536000	-0.074826000	H	3.446996000	2.849601000	-0.016033000
H	1.931795000	3.917694000	1.454865000	H	1.795512000	3.915853000	1.531910000
H	-0.289392000	2.874138000	1.878543000	H	-0.401071000	2.784716000	1.871696000
O	-3.213088000	-0.830024000	0.849643000	O	-3.132345000	-0.940302000	0.874493000
O	-0.832166000	-0.760749000	2.366983000	O	-0.743663000	-0.743845000	2.381078000
O	-3.002027000	1.878603000	0.460506000	O	-3.071880000	1.766428000	0.419374000
O	-2.441828000	1.299804000	-0.706199000	O	-2.468539000	1.209155000	-0.737401000
H	-1.493257000	-1.198839000	2.923594000	H	-1.404682000	-1.137052000	2.969149000
I	0.531440000	-2.693986000	2.300521000	Br	0.473734000	-2.528538000	2.312454000
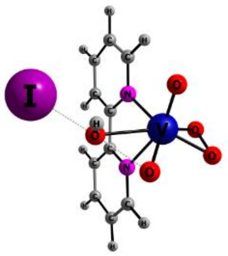				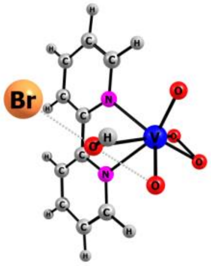			
Electronic energy: -8935.75690656 Eh				Electronic energy: -4428.26053100 Eh			

**A-I (bpy), A-Br (bpy), B-I (bpy) and B-Br (bpy) product (1a-bpy)**				**HOBr**			
Element and coordinates (Å, X, Y, Z)				Element and coordinates (Å, X, Y, Z)			
V	-2.331353000	0.294921000	0.649170000	O	0.356068000	-0.933160000	2.993614000
C	-1.424678000	-2.030489000	-1.114471000	H	-0.506658000	-0.608937000	2.704690000
C	-0.667207000	-2.856090000	-1.917573000	Br	0.311409000	-2.712060000	2.652294000
C	0.638936000	-2.498911000	-2.179666000				
C	1.131368000	-1.334773000	-1.628958000				
C	0.318124000	-0.556842000	-0.830082000				
C	0.794238000	0.694344000	-0.210777000				
C	2.073715000	1.189033000	-0.387267000				
C	2.420048000	2.371170000	0.235071000				
C	1.482839000	3.020092000	1.016436000				
C	0.229402000	2.451195000	1.141352000				
N	-0.952304000	-0.905460000	-0.576573000				
N	-0.102463000	1.320104000	0.538991000				
O	-1.887167000	0.121153000	2.220897000				
H	-2.455890000	-2.254950000	-0.877739000				
H	-1.109767000	-3.756468000	-2.321360000				
H	1.267478000	-3.119355000	-2.805906000				
H	2.151941000	-1.041032000	-1.824722000				
H	2.801955000	0.674362000	-0.997514000				
H	3.413792000	2.783409000	0.112863000				
H	1.707198000	3.948508000	1.524778000				
H	-0.545637000	2.912756000	1.743363000				
O	-3.522778000	-0.768367000	0.366786000				
O	-3.081310000	1.959608000	0.526715000				
O	-2.511298000	1.636235000	-0.754411000				
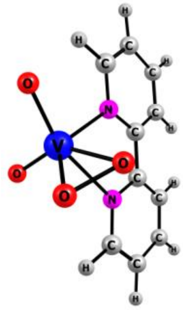				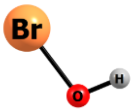			
Electronic energy: -1746.30037024 Eh				Electronic energy: -2681.99104005 Eh			

<table>
<tr><th>HOI</th><th>A-I (water) TS</th></tr>
<tr><td>Element and coordinates (Å, X, Y, Z)</td><td>Element and coordinates (Å, X, Y, Z)</td></tr>
<tr><td>O -0.544310000 -1.087432000 2.385852000
H -1.312762000 -1.245284000 2.943972000
I 0.482007000 -2.782653000 2.384577000</td><td>V -0.861013000 0.013505000 -0.301332000
O -1.305102000 0.424660000 1.326252000
O -0.763938000 -1.542433000 -0.586615000
O 0.375113000 -0.261689000 1.600107000
O -2.475717000 0.542909000 -0.991847000
O -1.378658000 0.958280000 -1.793461000
H -0.022986000 -1.089701000 1.916910000
O 0.982127000 0.808081000 -0.751962000
H 1.228985000 0.940461000 -1.673712000
H 1.747559000 0.428578000 -0.294237000
I 2.586883000 -1.226526000 1.960924000</td></tr>
<tr><td>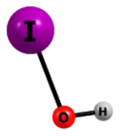
</td><td>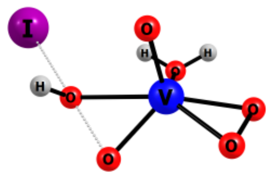
</td></tr>
<tr><td>Electronic energy: -7189.48716485 Eh</td><td>Electronic energy: -8516.587284254452 Eh</td></tr>
</table>

<table>
<tr><th>A-Br (water) TS</th><th>A-I (water), A-Br (water), B-I (water), B-Br (water) product (1a-w)</th></tr>
<tr><td>Element and coordinates (Å, X, Y, Z)</td><td>Element and coordinates (Å, X, Y, Z)</td></tr>
<tr><td>V -0.886712000 0.004909000 -0.299312000
O -1.316450000 0.379232000 1.328526000
O -0.804116000 -1.544992000 -0.637375000
O 0.420120000 -0.274713000 1.546675000
O -2.486321000 0.554669000 -1.019510000
O -1.364813000 1.070809000 -1.721155000
H 0.049854000 -1.053910000 1.990013000
O 0.984555000 0.735619000 -0.774418000
H 1.182977000 0.980453000 -1.684669000
H 1.736240000 0.235087000 -0.420985000
Br 2.484665000 -1.087161000 1.692207000</td><td>V -1.066757000 0.073266000 -0.387403000
O -1.249485000 0.573803000 1.145254000
O -0.727869000 -1.508704000 -0.506864000
O -2.598664000 0.427400000 -1.330032000
O -1.472017000 1.133264000 -1.872090000
O 0.858631000 0.840336000 -0.676303000
H 1.129618000 0.907751000 -1.598991000
H 1.564372000 0.373078000 -0.215108000</td></tr>
<tr><td>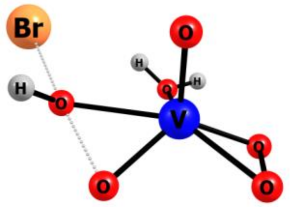
</td><td>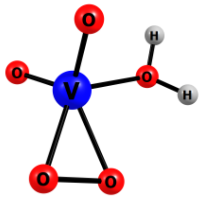
</td></tr>
<tr><td>Electronic energy: -4009.09905132 Eh</td><td>Electronic energy: -1327.15701700 Eh</td></tr>
</table>

B-I (bpy) TS1				B-Br (bpy) TS1			
Element and coordinates (Å, X, Y, Z)				Element and coordinates (Å, X, Y, Z)			
V	-1.902667000	0.050768000	0.912702000	V	-1.870004000	0.037565000	0.908728000
C	-1.291303000	-2.195298000	-1.070271000	C	-1.260202000	-2.209231000	-1.054017000
C	-0.564143000	-2.997425000	-1.922172000	C	-0.595746000	-2.968653000	-1.993758000
C	0.696924000	-2.591476000	-2.302884000	C	0.660955000	-2.565025000	-2.394450000
C	1.186946000	-1.408430000	-1.800527000	C	1.200651000	-1.419328000	-1.847153000
C	0.426510000	-0.671481000	-0.917894000	C	0.480553000	-0.707180000	-0.911220000
C	0.943635000	0.561216000	-0.301479000	C	0.989807000	0.524492000	-0.283008000
C	2.208224000	1.030447000	-0.587525000	C	2.210363000	1.087665000	-0.595763000
C	2.625959000	2.207987000	-0.006287000	C	2.592813000	2.248431000	0.047457000
C	1.774753000	2.873878000	0.852423000	C	1.753027000	2.803672000	0.992784000
C	0.538817000	2.310935000	1.120619000	C	0.553348000	2.170366000	1.256911000
N	-0.804697000	-1.059406000	-0.570346000	N	-0.736767000	-1.108195000	-0.522902000
N	0.137541000	1.179500000	0.556523000	N	0.184168000	1.065730000	0.626294000
O	-1.645275000	0.405566000	2.796069000	O	-1.697809000	0.538795000	2.775464000
H	-2.285955000	-2.458112000	-0.743192000	H	-2.247509000	-2.476311000	-0.701489000
H	-0.992374000	-3.922846000	-2.279251000	H	-1.069434000	-3.855927000	-2.390721000
H	1.297320000	-3.192522000	-2.971519000	H	1.215798000	-3.137229000	-3.126970000
H	2.169271000	-1.073771000	-2.087602000	H	2.183280000	-1.091929000	-2.153580000
H	2.870891000	0.491620000	-1.244236000	H	2.864940000	0.642127000	-1.330825000
H	3.611297000	2.602436000	-0.217623000	H	3.541684000	2.715086000	-0.185532000
H	2.052999000	3.801890000	1.333046000	H	2.012170000	3.708486000	1.526006000
H	-0.156167000	2.766153000	1.818069000	H	-0.143050000	2.539583000	2.001549000
O	-3.054633000	-1.028085000	0.958726000	O	-3.029199000	-1.038667000	0.950520000
O	-0.620231000	-0.880997000	2.124732000	O	-0.691282000	-0.858523000	2.238315000
O	-2.711738000	1.681247000	0.694692000	O	-2.682144000	1.643942000	0.566413000
O	-2.397638000	1.088570000	-0.542133000	O	-2.264711000	1.020672000	-0.622323000
H	-1.091061000	-1.650648000	2.463438000	H	-1.205839000	-1.560618000	2.651158000
I	-3.023202000	2.078284000	3.933901000	Br	-2.949861000	2.250205000	3.572114000
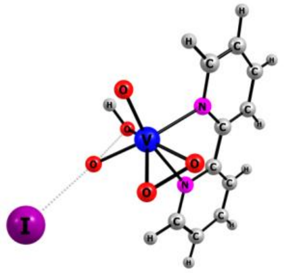				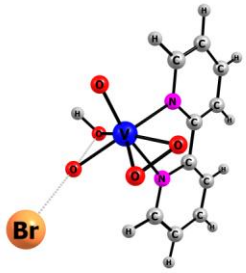			
Electronic energy: -8935.77334471 Eh				Electronic energy: -4428.27968207 Eh			

**B-I (bpy) Intermediate 2**				**B-Br (bpy) Intermediate 2**			
Element and coordinates (Å, X, Y, Z)				Element and coordinates (Å, X, Y, Z)			
V	-1.902824000	-0.027606000	0.995154000	V	-1.896472000	-0.038475000	0.979526000
C	-1.311135000	-2.200362000	-1.085582000	C	-1.280890000	-2.218741000	-1.074522000
C	-0.585589000	-2.997568000	-1.944819000	C	-0.614418000	-2.954729000	-2.032834000
C	0.676139000	-2.589124000	-2.319652000	C	0.636000000	-2.529826000	-2.430454000
C	1.166113000	-1.411183000	-1.804773000	C	1.166775000	-1.392522000	-1.858122000
C	0.406801000	-0.681269000	-0.911903000	C	0.443659000	-0.702970000	-0.905286000
C	0.937956000	0.547158000	-0.286251000	C	0.961458000	0.519743000	-0.258668000
C	2.207182000	1.002241000	-0.581638000	C	2.200330000	1.055372000	-0.553857000
C	2.643754000	2.177300000	-0.008581000	C	2.609320000	2.200155000	0.101587000
C	1.806928000	2.858725000	0.850650000	C	1.770411000	2.778595000	1.033000000
C	0.566990000	2.308668000	1.129769000	C	0.546270000	2.180234000	1.268245000
N	-0.822490000	-1.075140000	-0.571043000	N	-0.768055000	-1.123936000	-0.525287000
N	0.148735000	1.179286000	0.577688000	N	0.156875000	1.084341000	0.637633000
O	-1.915909000	0.587118000	2.887360000	O	-1.999744000	0.687404000	2.850378000
H	-2.307296000	-2.463930000	-0.761476000	H	-2.264483000	-2.502265000	-0.722721000
H	-1.014643000	-3.918802000	-2.312149000	H	-1.081102000	-3.838039000	-2.447179000
H	1.277661000	-3.184217000	-2.992834000	H	1.193026000	-3.078063000	-3.179641000
H	2.148565000	-1.076632000	-2.090932000	H	2.145508000	-1.052288000	-2.162499000
H	2.861180000	0.455845000	-1.240261000	H	2.854039000	0.599501000	-1.283209000
H	3.631763000	2.558200000	-0.231422000	H	3.576791000	2.636489000	-0.112917000
H	2.097521000	3.788357000	1.320802000	H	2.046005000	3.674208000	1.573723000
H	-0.120271000	2.778978000	1.825090000	H	-0.157601000	2.584385000	1.987658000
O	-3.153498000	-0.999478000	0.983982000	O	-3.138700000	-1.022158000	0.940685000
O	-0.604002000	-1.154520000	1.891342000	O	-0.672736000	-1.130392000	2.014789000
O	-2.654562000	1.646785000	0.675865000	O	-2.649761000	1.607043000	0.551217000
O	-2.338334000	0.997991000	-0.518003000	O	-2.243445000	0.934741000	-0.597833000
H	-0.675704000	-0.905602000	2.818403000	H	-0.705543000	-0.775715000	2.908088000
I	-3.189344000	1.815187000	3.692076000	Br	-2.982637000	2.090727000	3.374834000
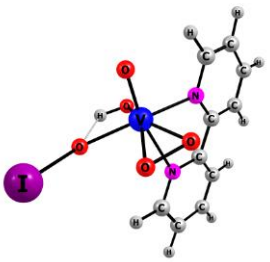				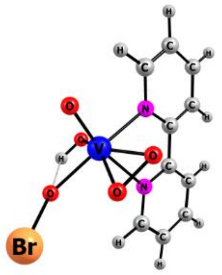			
Electronic energy: -8935.81577725 Eh				Electronic energy: -4428.31401358 Eh			

B-I (bpy) TS2				B-Br (bpy) TS2			
Element and coordinates (Å, X, Y, Z)				Element and coordinates (Å, X, Y, Z)			
V	-1.892338000	-0.005635000	0.866696000	V	-1.823334000	-0.042984000	0.876163000
C	-1.247921000	-2.219932000	-1.075225000	C	-1.201228000	-2.262083000	-1.072578000
C	-0.530945000	-3.034612000	-1.921321000	C	-0.555185000	-3.015431000	-2.028343000
C	0.730357000	-2.639312000	-2.310599000	C	0.679946000	-2.593289000	-2.472758000
C	1.225596000	-1.453376000	-1.823898000	C	1.218821000	-1.440000000	-1.942774000
C	0.475297000	-0.693830000	-0.950743000	C	0.522291000	-0.733046000	-0.985480000
C	1.016341000	0.556480000	-0.386516000	C	1.054142000	0.500624000	-0.378886000
C	2.283374000	0.991861000	-0.722620000	C	2.276509000	1.047905000	-0.718647000
C	2.728112000	2.189891000	-0.212171000	C	2.684482000	2.205972000	-0.087361000
C	1.903434000	2.910871000	0.625585000	C	1.864898000	2.778836000	0.865738000
C	0.664163000	2.382533000	0.944375000	C	0.659795000	2.163413000	1.147015000
N	-0.757279000	-1.075295000	-0.589279000	N	-0.682132000	-1.146995000	-0.559024000
N	0.235949000	1.229212000	0.449873000	N	0.268828000	1.058352000	0.533187000
O	-2.939931000	-0.057958000	3.438247000	O	-3.034912000	0.252717000	3.406041000
H	-2.242019000	-2.474748000	-0.742500000	H	-2.173805000	-2.544233000	-0.693925000
H	-0.970591000	-3.958938000	-2.267484000	H	-1.028128000	-3.911731000	-2.405271000
H	1.325255000	-3.248387000	-2.976708000	H	1.218865000	-3.155808000	-3.224439000
H	2.207282000	-1.130800000	-2.122532000	H	2.186648000	-1.099734000	-2.279794000
H	2.924862000	0.414317000	-1.365759000	H	2.915312000	0.592290000	-1.461268000
H	3.712841000	2.556654000	-0.468817000	H	3.636619000	2.657612000	-0.335674000
H	2.204459000	3.860508000	1.044968000	H	2.142835000	3.683564000	1.389738000
H	-0.011836000	2.897751000	1.617126000	H	-0.022429000	2.562305000	1.890002000
O	-3.073783000	-1.045713000	0.772399000	O	-3.003625000	-1.090076000	0.807282000
O	-0.792510000	-0.557964000	2.189296000	O	-0.831369000	-0.505079000	2.322111000
O	-2.654255000	1.625566000	0.795358000	O	-2.586979000	1.578089000	0.692186000
O	-2.270380000	1.238733000	-0.500967000	O	-2.149111000	1.135887000	-0.571868000
H	-1.513022000	-0.573493000	2.888492000	H	-1.571838000	-0.444894000	2.989543000
I	-2.740512000	1.315614000	4.804725000	Br	-2.665918000	1.767817000	4.299085000
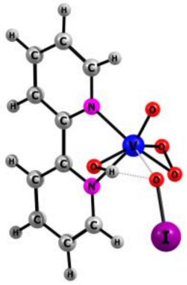				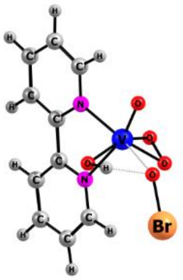			
Electronic energy: -8935.79241035 Eh				Electronic energy: -4428.29049925 Eh			

B-I (water) TS1				B-Br (water) TS1			
Element and coordinates (Å, X, Y, Z)				Element and coordinates (Å, X, Y, Z)			
V	-0.375786000	0.022779000	-0.114971000	V	-0.395941000	-0.017883000	0.008699000
O	-0.584911000	0.192690000	1.786474000	O	-0.610155000	0.214874000	1.898910000
O	-1.304100000	-1.180949000	-0.480798000	O	-1.275593000	-1.267572000	-0.321051000
O	0.948081000	-0.349559000	1.227882000	O	0.943796000	-0.348943000	1.357582000
O	-1.273152000	1.596455000	-0.339203000	O	-1.386881000	1.492221000	-0.256067000
O	-0.436203000	1.284352000	-1.429955000	O	-0.514333000	1.216602000	-1.328293000
H	0.976776000	-1.268398000	1.524186000	H	0.934180000	-1.246358000	1.712947000
O	1.233371000	-0.595109000	-1.367920000	O	1.244925000	-0.574898000	-1.210195000
H	1.309768000	-1.531385000	-1.580198000	H	1.675835000	-1.408384000	-0.987378000
H	1.257902000	-0.117166000	-2.205283000	H	1.057791000	-0.603201000	-2.155788000
I	-2.876552000	0.897609000	2.589852000	Br	-2.747749000	0.870868000	2.604046000
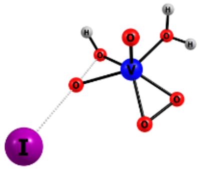				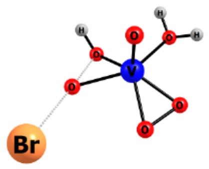			
Electronic energy: -8516.5978663 Eh				Electronic energy: -4009.11283816 Eh			

B-I (water) Intermediate 2				B-Br (water) Intermediate 2			
Element and coordinates (Å, X, Y, Z)				Element and coordinates (Å, X, Y, Z)			
V	-0.295606000	0.034276000	0.036689000	V	-0.537276000	0.144602000	-0.130248000
O	-1.028903000	0.067038000	1.857541000	O	-1.305755000	-0.029544000	1.676740000
O	-1.308767000	-1.034886000	-0.544292000	O	-1.571399000	-0.796892000	-0.872487000
O	1.173056000	-0.537698000	0.989029000	O	0.897829000	-0.586721000	0.786967000
O	-0.947974000	1.752378000	-0.066582000	O	-1.135354000	1.875305000	-0.030021000
O	-0.104598000	1.415537000	-1.140014000	O	-0.258528000	1.651187000	-1.105439000
H	0.975987000	-0.536151000	1.930763000	H	0.751253000	-0.612820000	1.736918000
O	1.197577000	-0.698250000	-1.582662000	O	0.974863000	-0.540918000	-1.700880000
H	1.196584000	-1.651904000	-1.704449000	H	1.566630000	-1.082915000	-1.164285000
H	0.881991000	-0.334095000	-2.419098000	H	0.558538000	-1.140093000	-2.329471000
I	-2.895664000	0.477817000	2.228000000	Br	-2.979266000	0.513798000	2.008745000
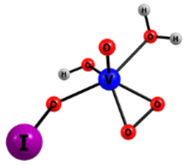				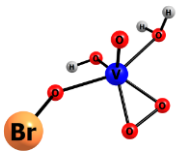			
Electronic energy: -8516.6603555 Eh				Electronic energy: -4009.16682898 Eh			

**B-I (water) TS2**				**B-Br (water) TS2**			
Element and coordinates (Å, X, Y, Z)				Element and coordinates (Å, X, Y, Z)			
V	-0.052807000	0.351106000	-0.196811000	V	-0.174066000	0.061314000	-0.319035000
O	-0.880656000	0.461100000	2.028023000	O	-1.297982000	-0.370315000	2.114106000
O	-1.324606000	-0.506802000	-0.638364000	O	-1.133834000	-1.105986000	-0.800792000
O	1.025549000	-0.321805000	0.977436000	O	0.844228000	-0.297483000	1.042680000
O	-0.404933000	2.132646000	-0.147040000	O	-1.069670000	1.621735000	-0.263323000
O	0.396677000	1.773101000	-1.257527000	O	-0.117905000	1.603462000	-1.310926000
H	0.154696000	-0.091063000	1.782924000	H	-0.003741000	-0.404038000	1.732879000
O	1.194543000	-0.673485000	-1.615677000	O	1.440855000	-0.431345000	-1.549866000
H	1.452073000	-1.510509000	-1.208724000	H	1.814746000	-1.303142000	-1.374396000
H	0.736401000	-0.904544000	-2.433423000	H	1.276966000	-0.385596000	-2.499288000
I	-2.296934000	-0.709743000	2.709184000	Br	-1.579595000	1.011393000	3.227963000
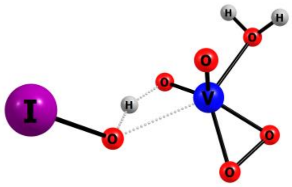				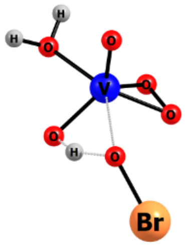			
Electronic energy: -8516.63469604 Eh				Electronic energy: -4009.13927478 Eh			

**Catalyst recovery: TS3 (bpy)**				**Catalyst recovery: TS3 (water)**			
Element and coordinates (Å, X, Y, Z)				Element and coordinates (Å, X, Y, Z)			
V	-1.664105000	0.339927000	0.680713000	V	-0.105676000	0.270057000	-0.392915000
C	-1.225300000	-2.200460000	-0.902342000	O	0.265258000	0.834559000	1.204552000
C	-0.638967000	-3.158015000	-1.701127000	O	0.047102000	-1.301339000	-0.695086000
C	0.641963000	-2.934923000	-2.160128000	O	-1.476105000	1.067769000	-1.270399000
C	1.283085000	-1.768898000	-1.799378000	O	-0.227746000	1.345290000	-1.869973000
C	0.641890000	-0.852890000	-0.991188000	O	2.002298000	0.674148000	-0.691039000
C	1.287201000	0.403962000	-0.568827000	H	2.536182000	0.028244000	-1.164837000
C	2.565501000	0.763009000	-0.952773000	H	2.362785000	0.742162000	0.202205000
C	3.082943000	1.961956000	-0.505344000	O	-1.721872000	-1.643249000	1.421171000
C	2.312665000	2.762795000	0.315334000	O	-1.750755000	-0.268550000	1.033590000
C	1.046585000	2.324088000	0.654995000	H	-0.782395000	0.282723000	1.485270000
N	-0.607399000	-1.073026000	-0.551313000	H	-1.149076000	-2.031812000	0.737461000
N	0.551211000	1.177645000	0.218121000				
O	-0.754647000	-0.017929000	2.166839000				
H	-2.229578000	-2.320513000	-0.520205000				
H	-1.192358000	-4.053396000	-1.948546000				
H	1.137598000	-3.661267000	-2.791575000				
H	2.287330000	-1.583227000	-2.150377000				
H	3.163777000	0.129734000	-1.591539000				
H	4.080554000	2.268630000	-0.793464000				
H	2.674681000	3.709621000	0.693499000				
H	0.398668000	2.907637000	1.299830000				
O	-2.960975000	-0.592971000	0.678740000				
O	-2.230121000	2.000768000	0.262919000				
O	-1.831209000	1.350656000	-0.923530000				
O	-2.771761000	0.971352000	3.036420000				
O	-3.843623000	0.023069000	3.214834000				
H	-3.845942000	-0.413818000	2.346170000				
H	-1.584459000	0.275652000	2.778589000				
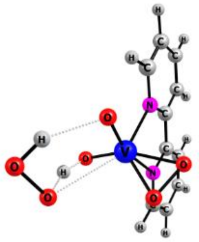				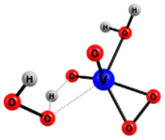			
Electronic energy: -1897.97844855 Eh				Electronic energy: -1478.83724961 Eh			

Catalyst recovery: Intermediate 3 (bpy) (3-bpy)				Catalyst recovery: Intermediate 3 (water) (3-w)			
Element and coordinates (Å, X, Y, Z)				Element and coordinates (Å, X, Y, Z)			
V	-1.880440000	0.088601000	0.415512000	V	-0.480359000	0.556672000	-0.797731000
C	-1.178063000	-2.359866000	-1.316563000	O	0.725691000	1.272491000	0.343379000
C	-0.462428000	-3.247111000	-2.094871000	O	0.173632000	-0.665940000	-1.559472000
C	0.855077000	-2.951915000	-2.377599000	O	-1.861858000	1.314567000	-1.750994000
C	1.399657000	-1.787838000	-1.876573000	O	-0.605373000	1.956665000	-1.957846000
C	0.623121000	-0.944481000	-1.106007000	O	-1.116816000	-1.000993000	1.109140000
C	1.152276000	0.315653000	-0.544132000	O	-1.856164000	-0.108790000	0.271037000
C	2.453681000	0.735084000	-0.745150000	H	0.587915000	2.177743000	0.637240000
C	2.869617000	1.925820000	-0.182579000	H	-1.326138000	-1.865929000	0.732126000
C	1.974451000	2.665223000	0.564334000				
C	0.690454000	2.174383000	0.715797000				
N	-0.651852000	-1.240726000	-0.833879000				
N	0.294413000	1.033780000	0.175069000				
O	-0.859108000	-0.933267000	1.683945000				
H	-2.214711000	-2.538389000	-1.060118000				
H	-0.942607000	-4.144170000	-2.461698000				
H	1.453359000	-3.621177000	-2.982909000				
H	2.430291000	-1.548345000	-2.093119000				
H	3.151191000	0.153077000	-1.329772000				
H	3.885254000	2.272884000	-0.325827000				
H	2.251939000	3.603231000	1.026676000				
H	-0.058936000	2.704020000	1.293498000				
O	-3.183771000	-0.818222000	0.350968000				
O	-2.443536000	1.731739000	-0.292062000				
O	-1.969099000	0.887672000	-1.294638000				
O	-2.269872000	1.249250000	1.981860000				
O	-2.299819000	0.632800000	3.274568000				
H	-3.178464000	0.235453000	3.303162000				
H	-1.131664000	-0.610477000	2.550799000				
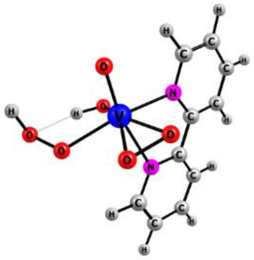				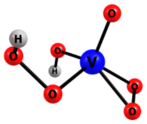			
Electronic energy: -1898.00786377 Eh				Electronic energy: -1402.36499509 Eh			

**Catalyst recovery: TS4 (bpy)**				**Catalyst recovery: TS4 (water)**			
Element and coordinates (Å, X, Y, Z)				Element and coordinates (Å, X, Y, Z)			
V	-1.347205000	0.178554000	0.297595000	V	-0.581423000	0.250147000	-0.542107000
C	-0.698485000	-2.336289000	-1.400819000	O	0.771904000	1.200953000	0.583831000
C	0.013895000	-3.256789000	-2.143000000	O	0.010765000	-0.868051000	-1.483331000
C	1.384808000	-3.120389000	-2.219164000	O	-2.005466000	1.000737000	-1.432074000
C	1.989523000	-2.073142000	-1.554019000	O	-0.827586000	1.796494000	-1.493599000
C	1.211100000	-1.196083000	-0.829391000	O	-0.379719000	-0.650281000	1.260872000
C	1.766471000	-0.058143000	-0.081378000	O	-1.689272000	-0.303720000	0.811495000
C	3.110423000	0.246178000	-0.043936000	H	0.617766000	2.139320000	0.723449000
C	3.529017000	1.336550000	0.693046000	H	0.251369000	0.397024000	1.319959000
C	2.592368000	2.086260000	1.375628000				
C	1.265569000	1.711677000	1.293587000				
N	-0.115088000	-1.330190000	-0.761162000				
N	0.864852000	0.670264000	0.576566000				
O	-0.246792000	-1.673337000	2.175153000				
H	-1.775067000	-2.391251000	-1.302548000				
H	-0.511228000	-4.059112000	-2.643181000				
H	1.977654000	-3.822871000	-2.791118000				
H	3.062014000	-1.953080000	-1.602432000				
H	3.835277000	-0.351084000	-0.578132000				
H	4.579014000	1.597053000	0.736293000				
H	2.870509000	2.946273000	1.969540000				
H	0.480713000	2.245029000	1.816239000				
O	-2.564484000	-0.837897000	0.250618000				
O	-1.903818000	1.770285000	-0.425902000				
O	-1.229988000	0.966051000	-1.372729000				
O	-1.787019000	0.120371000	2.935239000				
O	-1.555774000	1.049810000	1.883364000				
H	0.650304000	-1.398322000	2.376518000				
H	-0.868345000	-0.958530000	2.605577000				
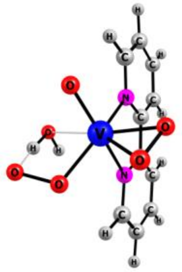				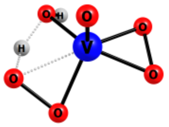			
Electronic energy: -1897.99435369 Eh				Electronic energy: -1402.34307808 Eh			

**Catalyst recovery: Product (bpy)**				**Catalyst recovery: Product (water)**			
Element and coordinates (Å, X, Y, Z)				Element and coordinates (Å, X, Y, Z)			
V	-1.841741000	0.194351000	0.614698000	V	-0.725830000	0.171030000	-0.420706000
C	-1.162648000	-2.232402000	-1.103016000	O	0.893239000	1.155413000	0.550949000
C	-0.464941000	-3.116957000	-1.898343000	O	0.169938000	-0.733841000	-1.362510000
C	0.843578000	-2.818860000	-2.217803000	O	-2.045809000	0.970697000	-1.455760000
C	1.398940000	-1.653770000	-1.731940000	O	-0.974975000	1.846914000	-1.126972000
C	0.642419000	-0.812779000	-0.940819000	O	-0.926535000	-0.574521000	1.244815000
C	1.179363000	0.447083000	-0.391360000	O	-2.010033000	-0.902599000	0.384215000
C	2.470234000	0.881244000	-0.624660000	H	0.921546000	2.108857000	0.413703000
C	2.884077000	2.074741000	-0.066796000	H	0.933592000	1.014774000	1.503473000
C	1.997814000	2.798078000	0.706586000				
C	0.724276000	2.291023000	0.889304000				
N	-0.627956000	-1.107549000	-0.633036000				
N	0.330002000	1.147172000	0.353422000				
O	-0.908275000	-0.550237000	2.076653000				
H	-2.191074000	-2.411992000	-0.818520000				
H	-0.953005000	-4.015439000	-2.250601000				
H	1.426355000	-3.487039000	-2.839135000				
H	2.422632000	-1.409939000	-1.974973000				
H	3.158453000	0.309769000	-1.230473000				
H	3.890680000	2.436914000	-0.234109000				
H	2.274432000	3.738004000	1.165586000				
H	-0.016282000	2.810882000	1.485634000				
O	-3.117707000	-0.760908000	0.533585000				
O	-2.484070000	1.899812000	0.265773000				
O	-2.066466000	1.257146000	-0.928467000				
O	-1.653519000	0.609653000	2.413424000				
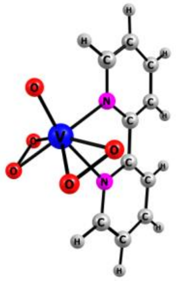				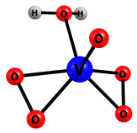			
Electronic energy: -1821.52498202 Eh				Electronic energy: -1402.37021135 Eh			

**Catalyst recovery: water**	**Catalyst recovery: hydrogen peroxide**
Element and coordinates (Å, X, Y, Z)	Element and coordinates (Å, X, Y, Z)
O -2.487462000 0.313391000 3.593877000<br>H -3.386344000 0.090917000 3.338790000<br>H -1.936223000 -0.266167000 3.062082000	O -1.692347000 1.677409000 2.702972000<br>O -1.888870000 0.840643000 3.835153000<br>H -1.602054000 1.407225000 4.562765000<br>H -2.581546000 2.016939000 2.540002000
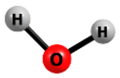	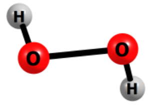
Electronic energy: -76.50308547 Eh	Electronic energy: -151.69005607 Eh

**Catalyst recovery: iodide**	**Catalyst recovery: bromide**
Element and coordinates (Å, X, Y, Z)	Element and coordinates (Å, X, Y, Z)
I 0.000000000 0.000000000 0.000000000	Br 0.000000000 0.000000000 0.000000000
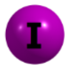	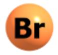
Electronic energy: -7113.81677209 Eh	Electronic energy: -2606.33296086 Eh
